# A study to better understand under-utilization of laboratory tests for antenatal care in Senegal

**DOI:** 10.1371/journal.pone.0225710

**Published:** 2020-01-09

**Authors:** Anna Helena van’t Hoog, Aicha Sarr, Winny Koster, Louis Delorme, Souleymane Diallo, Jean Sakande, Constance Schultsz, Christophe Longuet, Ahmad Iyane Sow, Pascale Ondoa

**Affiliations:** 1 Department of Global Health, Amsterdam University Medical Centers, Amsterdam, The Netherlands; 2 Amsterdam Institute for Global Health and Development, Amsterdam, The Netherlands; 3 Directorate of Laboratory Services, Ministry of Public Health and Social Welfare, Dakar, Senegal; 4 Fondation Mérieux, Dakar, Sénégal; 5 Amsterdam Institute for Social Science Research, University of Amsterdam, Amsterdam, The Netherlands; 6 Fondation Mérieux, Lyon, France; 7 Centre d’Infectiologie Charles Mérieux, Bamako, Mali; 8 Fondation Mérieux, Ougadougou, Burkina Faso; 9 Direction of Health Laboratories of Burkina Faso, Ougadougou, Burkina Faso; Liverpool School of Tropical Medicine, UNITED KINGDOM

## Abstract

**Objective:**

To better understand factors contributing to underutilization of laboratory services for health care delivery in sub-Saharan Africa, we conducted a study in Senegalese Antenatal Care clinics (ANC) and laboratories to determine the extent of underutilization, contributing factors, and bottlenecks in the cascade of care from first ANC visit, test uptake, to availability of test results and appropriate clinical management.

**Methods:**

At 16 health facilities, pregnant women attending for their first ANC visit were consecutively recruited and information was prospectively collected on the request, execution, results and clinical management of seven nationally recommended laboratory screening tests for normal pregnancy: hemoglobin concentration (Hb), syphilis serology, HIV serology, determination of proteinuria (PU), determination of blood group and Rhesus factor, Emmel test to detect sickle cell disease, and glycaemia. Health facility staff were interviewed on human resource capacity, management of the ANC and the laboratory, and availability and use of guidelines.

**Results:**

Of 1246 ANC attendants, 400 (32%) had complete results. Completeness varied between facilities from 0–99%. In multilevel logistic regression analysis of women nested in facilities, complete uptake was lower if women started ANC later in pregnancy; very low in rural ANC attendants who ever delivered compared to urban primigravidae (OR 0.064; 95%CI 0.00–0.52); and higher if the facility routinely recommended all seven tests. In the cascade from test request to clinical management, the most frequent bottleneck was non-execution of requested tests, while unavailability of results for executed test was uncommon (<2%). Overall, of 525 abnormal test results 97(18%) had a record of adequate clinical management.

**Conclusion:**

Our study illustrates challenges to test uptake even when laboratory testing capacity is in place, with large differences between facilities, and underscores the importance of management, policy, and the importance of considering local context in order to improve service delivery to expectant mothers.

## Introduction

It is increasingly recognized that inadequate laboratory infrastructure in Low- and Middle-Income Countries (LMICs) hampers the contribution of laboratory diagnostics to evidence-based clinical care[1]. This recognition has resulted in laboratory capacity building initiatives[2–4], and in the development of simple, rapid, and affordable point-of-care tests (POCTs), which do not require electricity, a laboratory, or highly trained staff[5]. However, other factors also contribute to underutilization. Antenatal care (ANC) provides a relevant model to study factors contributing to utilization of laboratory testing for health care delivery.

The aims of ANC include prevention and alleviation or management of health conditions that are known to have an unfavorable outcome on pregnancy[6]. ANC involves laboratory screening of infectious diseases and conditions like anemia and pre-eclampsia that are associated with increased maternal and/or newborn morbidity and mortality. The World Health Organization (WHO) has defined a minimum ANC package (focused ANC) for normal pregnancies, which is based on four goal-oriented visits[7]. The increase in ANC coverage is considered a success-story in Africa with 80% of women in sub-Saharan Africa receiving at least one ANC visit, however 52% completing 4 visits[8]. Relevant indicators for the utilization of laboratory screening tests in the context of ANC are scarce, with the exception of syphilis for which 59% of pregnant women in sub-Saharan Africa were tested in 2010 [9].

The WHO guidelines for focused ANC recommend testing for hemoglobin concentration, syphilis, HIV infection, proteinuria, blood group/Rhesus factors determination and bacteriuria [7], tests for which testing capacity is widely available in LMICs. The first four are recommended as a minimum for LMICs [7]. Countries are expected to adapt the WHO recommendations to their own epidemiological profile and priorities and implement national ANC guidelines with country specific detailing of the essential components of ANC.

We conducted a transdisciplinary mixed methods study with the overall aim to obtain a better understanding of the underutilization of the laboratory for antenatal care in three West African countries, Senegal, Burkina Faso and Mali (the study was called SociaLab [10]). Here we report on Senegal and describe the extent of underutilization of laboratory tests, contributing factors, and bottlenecks in the cascade of care from first ANC visit, test uptake, to availability of test results and appropriate clinical management. A qualitative analysis to further understand reasons underlying these patterns, and the activities to translate study findings into practice are reported elsewhere [10]. With 96% of pregnant women in 2014 attended ANC at least once, ANC coverage is high in Senegal compared to sub-Saharan Africa (SSA) in general, and 48% had 4 ANC visits, which is slightly lower [8]. Testing capacity for ANC screening tests is widely available. Maternal mortality was estimated at 315 death /10^5^ live births in 2015, declining from 427 in a decade [11][12] and neonatal mortality estimates declined from 30.8 to 21.3 per 1000 life births [11]. Syphilis testing in ANC Senegal was reported to be 5% in 2013, 11% in 2014, and 30% in 2015 [13].

## Methods

### Setting and laboratory tests

Senegal is a low-income country of West Africa with a gross national income of USD 950 per capita, a population of 15.4 million of whom 47% live below the poverty line. The adult literacy rate is 43% [14].

The policy of the Senegalese ministry of Health on ANC laboratory tests is in line with WHO recommendations [7]. National guidelines recommend seven mandatory tests for normal pregnancy [15]: hemoglobin concentration (Hb), syphilis serology, human immunodeficiency virus (HIV) serology, determination of proteinuria (PU), determination of blood group and Rhesus factor, Emmel test to detect sickle cell disease, and glycaemia. The national guidelines include normal ranges and recommended clinical management in case of critical results (Table 1). The seven recommended tests are to be requested and completed at first ANC visit. During following visits, repeat testing is on indication only (such as repeated proteinuria or glycemia in case of initially high values). Tests with lifelong result validity such as the blood group/Rhesus factor determination and the Emmel test are not supposed to be repeated once the result is known, but their status should be recorded on the ANC cards. The costs of the ANC consultation and the laboratory tests are covered by the patients, except for the rapid test for HIV serology, which is provided for free. Tests available as rapid format (HIV serology and proteinuria) can be delivered either at the laboratory facility or at the point of ANC, at the discretion of the health facility.

**Table 1:** Normal ranges for the seven recommended tests and criteria to assess clinical management.

Test (Reference)	Normal Ranges	Criteria to assess management if abnormal result
**Glycemia (1)**	>0.92 g/L Abnormal; ≤0.92 g/L Normal	OK: At least confirm the hyperglycemia with additional test(s), with or without dietary advise and exercise or refer to doctor, whether glycemia is confirmed or not.
		Partly OK: give dietary and exercise without referring to the doctor.
		Not OK: no clinical management recorded.
**Hemoglobin (2)**	<7g/dl : severe anemia	OK: Fe + Folate + referral for blood transfusion
		Partly OK: Referral for blood transfusion only
		Not OK: Fe and/or Folate and/or nothing
	Between 7 and 11g/dl : moderate anemia*	OK: Fe + Folate
		Partly OK: Fe only
		Not OK: Folate only, or no prescription
	>11g/dl Normal	
**Proteinuria**	Positive / Negative / Trace	OK: request microscopy and '24 hour proteinuria' OR refer to doctor is confirmed.
		Partly OK: Either microscopy or '24 hour proteinuria'.
		Not OK: nothing done or only dietary advise.
**Semi quantitative HIV test**	Positive / Negative	OK: should contain at least referral to social services, or directly to PMTCT. May include laboratory confirmation.
	(further quantification of HIV-type may be done if positive)	
**Syphilis RPR**	Positive (RPR +) / Negative (RPR -)	OK: RPR+ confirmed with TPHA.
** **	If RPR+ a TPHA is indicated	Partly OK: RPR+ without TPHA confirmation, but treatment prescribed.
** **		Not OK: no clinical management recorded.
**Syphilis TPHA**	Negative or titer 1/16: Negative	
	Titer 1/64 or 1/256: Positive	OK: at least benzylpenicillin prescribed.
**Emmel test**	Positive / Negative	OK: at least confirmation with electrophoresis with or without prescription of Fe.
		Partly OK: only prescription of Fe.
		Not OK: none of the above. Only dietary advice.
**Blood group/Rhesus**	Rhesus positive or negative	OK: prescription of serum anti-D and counselling with or without referral to the doctor.
		Partly OK: referral to doctor without prescribing the serum.
		Not OK: no clinical management recorded.

Fe = Ferrous Sulfate; RPR = Rapid Plasma Reagent; TPHA = Treponema pallidum hemagglutination assay

Reference: (1) OMS (2) PHP = Ministère de la Santé, de l’Hygiène Publique et de la Prévention, Protocoles de services de SR au Sénégal. 2014 [15]

*Same applies if Hemoglobin result is unavailable

During the first ANC visit, a pregnant woman is usually provided with a test request slip, which she is expected to take to the laboratory herself, once she is ready to pay and have her sample(s) collected. The laboratory hands over the results to the pregnant woman, usually on the same day or the day after, to share with the ANC staff during the next ANC consultation (usually some two months after the first visit). In case of abnormal results, the woman needs to be referred to ANC immediately. Results of rapid tests executed at the ANC are immediately available.

Within the Senegalese health system, the Directorate of Reproductive Health and Infant Survival (DSRSE) is responsible for the clinical component of ANC, while the Directorate of Laboratory services (DL) is responsible for laboratory testing. Screening for HIV infection is part of the Prevention of Mother-to-child Transmission (PMTCT) component of the national HIV program. Laboratory capacity for the seven recommended ANC screening test is in place as part of the tier-specific testing package at all health facilities of levels 2 (Etablissement Public de Santé: EPS) and 3 (Centres de Santé: CS) of the health care pyramid. Rapid testing for HIV and albuminuria are also supported at the level of health posts (Postes de Santé: PS), which do not host a laboratory. Laboratories at health facilities serve ANC women (and other persons) seeking clinical services at the same facility and as well as those referred directly to the laboratory by smaller public or private facilities with no access to laboratory testing.

### Study design

For the SociaLab study we targeted health facilities at the intermediate level of the Senegalese public health care system (level 2 and 3), providing services to more than 500 ANC clients per year. Of 93 level 2 and 3 facilities, we purposively selected 16, balancing geographic representation of Senegal’s 14 administrative regions and accessibility.

Pregnant women attending for their first ANC visit (ANC-W) were eligible to participate. From February 2014, at each study facility one or two midwives (depending on staff availability) were trained on the study procedures. The study-midwifes recruited consecutively among women assigned to their clinic hours, until the facility close-out visit in July/August 2014 or a maximum of 120 was reached, whatever came first. The sample size assumed on average 500 clients per year per facility (based on records from previous year) and the ability to determine a 50% test uptake (of 7 tests) with a 95% confidence interval of ± 3%, requiring a minimum sample size of 947 women. We allowed the 16 facilities to enroll 50% more women in anticipation of potential loss to follow-up. In addition to ANC-W, we included pregnant women who were referred to the laboratory from health posts and private practitioners for ANC laboratory tests (LAB-W) during the same period, up to a maximum of 50 per facility.

### Data handling and statistical analysis

Of the participating ANC-W, we prospectively transferred routinely collected information on age, pregnancy status, test requests, execution, results and clinical management (if applicable) to individual paper study cards (S1 Supporting File). For the study participant that were referred to the laboratory from smaller clinical facilities (for LAB-W), the same study cards were used, but only information on test request and execution were collected.

We obtained information about the selected health facilities during one-week visits to each health facility, between July 2013 and August 2014. One researcher (AS) conducted surveys based on 3 structured questionnaires addressing general characteristics, staffing/human resource, availability and use of guidelines, management and organization of the laboratory and the ANC services (S2 Supporting File). The three questionnaires were designed in such a way that they targeted 1) the ANC in charge; 2) the laboratory in-charge and 3) the finance administrator of the health facility.

Two additional research team members observed the ANC and laboratory premises to collect data about infrastructure and cleanliness. Information of the average waiting time of ANC visits was based on observing 10 random women attending the ANC clinic on the first day of the facility visit by the study team. In total each facility was described by one set of responses to the three different questionnaires. Two trained research assistants entered and stored both women and facility data in electronic databases (Open Clinica version 3.1. Copyright © OpenClinica LLC and collaborators, Waltham, MA, USA, www.OpenClinica.com). All entries were checked for completeness and quality against the source documents.

#### Definitions

*Test uptake*: the percentage of women attending for their first ANC visit who obtained complete results for all seven recommended ANC laboratory tests.

*Test utilization*: the percentage of women attending for their first ANC visit who, from the recordings on the cards, received appropriate clinical care in case of critical (out of range or abnormal) laboratory test results. For this outcome two investigators (PO, AS) reviewed the recorded information on clinical management of all participants possessing test results that were not negative or that fell outside the normal range. They categorized clinical management recordings as ‘appropriate’ when fully in accordance with the guidelines [15], ‘partly appropriate’ when necessary preventive or complementary measures to the treatment were missing or as ‘not appropriate’ in case no treatment or inadequate treatment was prescribed.

We recoded parity as ‘ever delivered’, implying experience with pregnancy and childbirth, versus ‘never delivered (nulliparous)’, assuming inexperience. We calculated turn-around times of test request, execution, recording of results, and return with results at the ANC for all tests combined, using the dates that were recorded on the cards.

We constructed cascades of care for ANC-W, per test and per facility, reflecting the total numbers of women attending for first ANC visit, with test request, test execution, available result, result abnormal or otherwise indicative of the need for further action, and adequate clinical management.

We summarized the data on health facilities by question and used the WHO health systems building blocks framework [16] to categorize questions as: Service delivery and quality; Governance and leadership; Information and research; Infrastructure, equipment and supplies; Human resources; Health care financing (Table 2). Two investigators (AH, PO) then, for each building block, assessed the suitability of all questions for inclusion as facility level co-variates in the analysis of factors associated with test uptake. We discarded questions that we considered too remote or otherwise irrelevant, had incomplete responses, lack of any variability in responses based on eyeballing the data. Table 2 shows the resulting selection of variables, their categories and distributions.

**Table 2:** Characteristics of the 16 health facilities and their laboratories, categorized by the six domains of the WHO health system building blocks. As described in the methods, parameters from the health facility assessment questionnaires were assessed for completeness and relevance for inclusion in further analysis. The building blocks were slightly adapted to the ANC-laboratory setting. The columns at the left show the distribution of the characteristics among the 16 facilities.

Domain / Questions			n	%
**Service delivery and quality**				
*All component regarding the delivery of effective*, *safe*, *quality ANC (including laboratory testing) to women attending ANC consultation*, *when and where needed*, *with minimum waste of resources*.				
	Are all 7 recommended ANC tests routinely requested?			
		Yes	11	69%
		No	5	31%
	Waiting time in ANC, in minutes (median, IQR)*		101 (66–141)	
		≤ 60 minutes	4	25%
		61–120 minutes	6	37,5%
		> 120 minutes	6	37,5%
	The complexity a woman encounters in getting her 7 tests executed and obtain results			
		I. 3 moments at 2 places (1 RT at ANC; 1 RT at lab; 1 or more non-rapid /not same-day tests at lab)	5	31%
		II. 2 moments at 2 places (2 RT at ANC; all other non-rapid /not same-day tests at lab)	9	56%
		III. 2 moments at 1 place (2 RT at lab; all other non-rapid /not same-day tests at lab)	2	13%
	Turnaround time (TaT) in days, from test request to execution, median of ANC-W at the respective facility (median, IQR)		6.3 (2–28)	
**Governance and leadership**				
*Leadership and governance involves ensuring that strategic policy frameworks exist (including test guidelines*, *price lists*, *staffing norms*, *etc*.*) and are combined with effective oversight*, *coalition-building*, *the provision of appropriate regulations and incentives*, *attention to system-design*, *and accountability at both system and facility level*.				
	Total hours per day that the lab sample collection is open			
		2–2.5 hours	3	19%
		3–4 hours	8	50%
		5–8.5 hours	5	31%
**Information and research**				
*Issues influencing the production*, *analysis*, *dissemination and use of reliable and timely information on health determinants*, *health systems performance and health status*. *This includes the technical and human aspect of the laboratory-clinical interface*.				
	Availability of standardized test request and result forms			
		request and result forms both available	1	6%
		request form unavailable; result form available	10	61%
		request and results forms both unavailable	5	33%
**Infrastructure, equipment and supplies**				
*Issues influencing equitable access to ANC and ANC testing technologies of assured quality*, *safety*, *efficacy and cost-effectiveness*, *and their scientifically sound and cost-effective use*. *Such as building*, *equipment*, *furniture and test kits*				
	Were there stockouts of reagents in 2012?			
		Yes	10	62,5%
		No	6	37,5%
	Were any of the laboratory equipment (for ANC tests) broken down at the time of observation?			
		Yes; either the biochemistry machine or the hemato analyzer or both were broken down.	4	25%
		No	12	75%
**Human resources**				
*A well-performing workforce that works in ways that are responsive*, *fair and efficient to achieve the best ANC outcomes possible*, *given available resources and circumstances*. *I*.*e*. *there are sufficient numbers and mix of mid wives*, *lab staff; they are competent; knowledgeable about the procedure*, *responsive and productive*. *Workforce engage in an effective lab-clinic interface*. *Workforce is properly trained*, *remunerated*, *and motivated*. *Workforce uses a client-centered approach*.				
	Shortage of midwives, based on the decree # 2009–521 of the MoH Senegal (1)			
		Yes	5	31%
		No	11	69%
**Health care financing**				
*Issues around raising adequate funds for health*, *in ways that ensure patients can use ANC services*, *that are affordable*. *Includes financing of the facility so that services can be provided in an affordable way*				
	Price of full set of 7 recommended ANC laboratory tests, in XAF (median, IQR)		9350 (7850–10750)**	

WHO = World Health Organization ANC = Antenatal Care RT = rapid test

ANC-W = Pregnant women in the study who attended the facility for their first ANC visit

*Obtained by observing the waiting time of 10 women attending ANC at each facility.

**Euro 14.25 (11.97–16.39) on Feb 1st 2014; oanda.com—XAF to Euro

(1) Ministère de la Santé, de la Prévention et de L’hygiène Publique. Décret n° 2009–521 du 4 juin 2009

We examined associations between test uptake (i.e. attainment of the complete set of seven recommended ANC test results among ANC-W) as an outcome and all available women and facility-related co-variates in univariate analysis and in a multilevel mixed model using the STATA XTMELOGIT command, to accommodate the hierarchical nature of the data [17]. We first modeled women nested in facilities without co-variates and added women level co-variates as fixed effects (parity trimester of first visit, women’s age) based on the magnitude of the association in crude analysis. We then added urban vs. rural (as our primary level 2 variable of primary interest) as a fixed effect and examined interactions between this variable and level 1 co-variates. Lastly, we added type of facility (hospital vs. health centre) and the facility level co-variates as listed in Table 2 and representing the WHO health system building blocks, one-by-one as fixed effects. Likelihood ratio tests (LRT) were used to compare models, and to explore if any of the fixed effects improved model fit. We obtained intra-class correlation coefficients (ICCs) with the Stata ESTAT command. Records with missing values for age and/or trimester of the pregnancy (n = 63) were excluded from the regression analysis. STATA version 12.1 (StataCorp LP, College Station, Texas, USA)[18] was used for all analyses.

### Ethical consideration

Ethical approval for the SOCIALAB study was granted by the Senegalese *Comité National d’Ethique pour la Recherche en Santé* (CNERS, protocol number SEN13/09). All study participants provided oral informed consent to preserve the anonymity of data collection and as per approval of the ethical review board. Study participants were provided a study number and all data were anonymized. Test request and results of HIV and syphilis testing were coded in such a way that the disease screened could not be identified.

## Results

### Facilities and pregnant women

Of the 16 selected facilities three were hospitals and 13 health centers, of which four were in rural areas, as defined by the Senegalese administrative criteria (Table 3). The number of women attending for ANC visit in 2012 ranged between 517 and 2917. The price of the full ANC package ranged between XAF 7,150 and 13,000 (Euro 10.9–19.8). At two facilities all seven ANC tests were conducted in the laboratory. At 14 facilities HIV serology rapid tests were conducted in the ANC and at nine of those proteinuria rapid tests as well. In addition to nurses, midwives and laboratory technicians, one of the four rural facilities had a gynaecologist, and five of the 12 urban facilities had both gyneacologist(s) and specialized laboratory staff. Additional health facility characteristics categorized by health system building block are summarized in Table 2.

**Table 3:** Characteristics and number of participants in the 16 selected health facilities.

	Type of Facility	Type of Area	# women attending ANC for 1st visit 2012	# participating ANC-women	# participating LAB-women	Average # of available results (out of 7) per ANC woman	% of ANC women with all 7 test results available.
A	Health Centre	Urban	736	83	25	6,2	38,6%
B	Health Centre	Rural	499	72	26	3,3	0,0%
C	Health Centre	Urban	559	64	29	2,8	15,6%
D	Health Centre	Urban	1299	86	27	5,1	60,5%
E	Health Centre	Rural	569	74	26	2,7	10,8%
F	Health Centre	Urban	653	80	26	4,0	37,5%
G	Health Centre	Urban	1198	74	24	3,2	24,3%
H	Health Centre	Urban	1822	76	26	6,6	71,1%
I	Health Centre	Rural	1002	78	26	4,6	24,4%
J	Health Centre	Rural	2917	77	26	1,7	0,0%
K	Hospital	Urban	736	87	27	5,3	33,3%
L	Hospital	Urban	637	75	30	7,0	98,7%
M	Hospital	Urban	517	78	48	2,9	3,8%
N	Hospital	Urban	734	74	25	2,6	4,1%
O	Health Centre	Urban	2297	97	30	2,6	1,0%
P	Health Centre	Urban	1564	71	25	6,9	94,4%

ANC = Antenatal clinic

ANC-women are pregnant women attending the facility for their first ANC visit

LAB-women are referred to the laboratory from health posts and private practitioners for ANC laboratory tests

In total 1692 women participated in the study, per facility a median of 76.5 ANC-W (range 64–97) and 26 LAB-W (range 24–48) (Table 3). Of the participants, 1246 (74%) were ANC-W of whom 438 (35%) were nulliparous and 754 (61%) were in the first trimester of their pregnancy (Table 4). LAB-W (n = 446) had a similar age distribution, and 191 (42.8%) were nulliparous. Compared to ANC-W they presented at the lab at a more advanced stage of the pregnancy (34% in the first trimester) and were less often attended by specialized ANC staff (i.e mid-wife or gynecologist, 75%) compared to ANC-W (99%).

**Table 4:** Characteristics of 1692 pregnant women who participated in the study.

Entry point	ANC n = 1246 (74%)		LAB n = 446		
	N	column %	n	column %	Chi-sq
Age category					p = 0.177
13–19	210	16,9%	95	21,3%	
20–24	375	30,1%	127	28,5%	
25–29	294	23,6%	94	21,1%	
30–34	201	16,1%	74	16,6%	
35–39	117	9,4%	33	7,4%	
missing	49	3,9%	23	5,2%	
Ever delivered					p = 0.002
No	438	35,2%	191	42,8%	
Yes	792	63,6%	243	54,5%	
missing	16	1,3%	12	2,7%	
Trimester					p<0.001
First	754	60,5%	152	34,1%	
Second	415	33,3%	206	46,2%	
Third	63	5,1%	61	13,7%	
missing	14	1,1%	27	6,1%	
Type of Facility					p = 0.104
Health Centre	932	74,8%	316	70,9%	
Hospital	314	25,2%	130	29,1%	
Qualification ANC staff					p<0.001
midwife	1.234	99,0%	313	70,2%	
gyn/physician	2	0,2%	22	4,9%	
nurse	7	0,6%	86	19,3%	
not specified	3	0,2%	25	5,6%	

ANC = Antenatal clinic LAB = laboratory

### ANC test uptake and determinants

Of the seven recommended tests, ANC-W on average had 4.2 results available (sd 2.7), and 400 (32%) had complete results for the set, implying optimal test uptake. Completeness varied widely between facilities (Table 3), from 94% and 99% at the highest end, to 0% at two facilities, where women on average had 1.7 and 3.3 test results.

In multilevel logistic regression analysis, ANC-W had half the odds of a complete set of 7 results if they made their first ANC visit in the third trimester (compared to first) or had ever delivered. Women’s age was not associated with availability of complete results. Facility-level variation (as a random effect) explained most of the variation in the data (ICC 72%). The addition of facility-level characteristics to the model as fixed effects explained some of this variation (ICC 58%). The final model (Table 5) suggests that women attending rural facilities were at lower odds of complete results, but this interacted with parity, such that for women who ever delivered and attended ANC at rural health facilities, the odds of complete results were very low compared to urban primigravidae (OR 0.064; 95%CI 0.00–0.52). At urban facilities women who ever delivered had slightly reduced odds of complete results compared to urban primigravidae (OR 0.72; 95%CI 0.49–1.06). Whether a facility was a hospital or health centre had no significant effect. Of the facility-level building block questions listed in Table 2, two improved the model. Routine recommendation of all 7 tests increased the odds, and a larger number of daily opening hours of the lab for sample collection decreased the odds of complete results, however, the 95%CIs of the effect sizes were wide.

**Table 5:** Factors associated with the availability of all results for the 7 recommended ANC laboratory tests, obtained in a multilevel logistic regression model of 1183 women nested in 16 health facilities.

7 Test results available	YES (n, %)		NO (n, %)		Final multilevel model	
					OR	95% CI
**Total**	400	32%	846	68%	** **	
Ever delivered(1) X Area(2)						
Urban, never delivered	147	45%	183	55%	1	
Urban, ever delivered	213	38%	348	62%	0.72	0.49; 1.06
Rural, never delivered	17	18%	77	82%	0.37	0.05; 2.88
Rural, ever delivered	9	5%	189	95%	0.06	0.008; 0.52
Trimester(1)						
First	257	35%	472	65%		
Second	116	29%	278	71%	0.68	0.47; 0.99
Third	13	22%	47	78%	0.49	0.22; 1.09
All 7 tests routinely recommended (Building block: Service delivery and Quality)(2)						
Yes	348	44%	448	56%	10.8	1.67; 69.9
No	38	10%	349	90%	1	
Total hours per day that the lab sample collection is open (Building block: Governance and Leadership) (2)						
2–2.5 hours	152	67%	76	33%	1	
3–4 hours	222	38%	368	62%	0.522	0.068; 4.0
5–8.5 hours	12	3%	353	97%	0.009	0.001; 0.10

OR = odds ratio 95%CI = 95% Confidence Interval

(1) = level 1 variable (women); (2) = level 2 variable (health facilities)

### Bottlenecks

**Fig 1:**
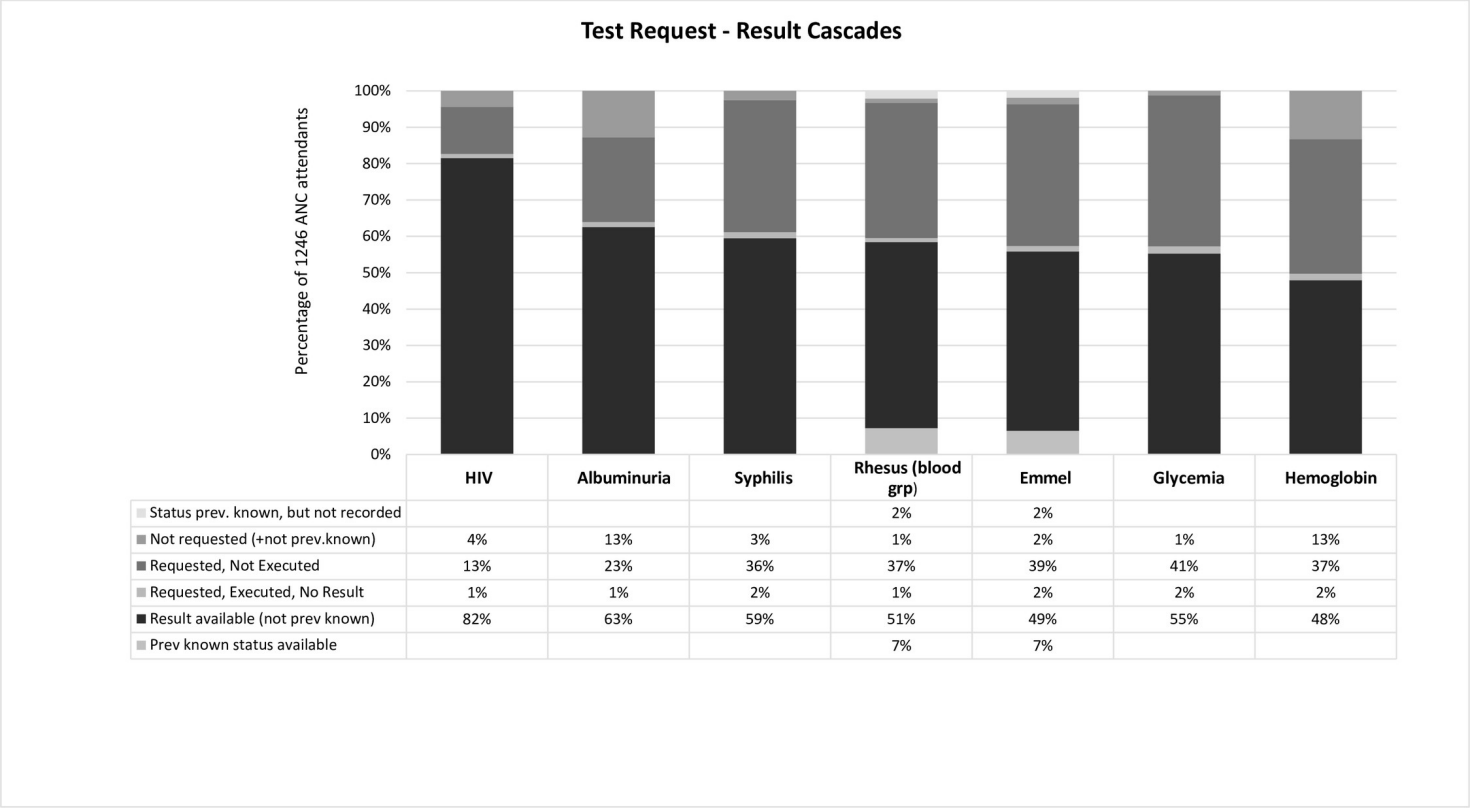
Bottlenecks in the cascade from test request to result for the seven recommended ANC laboratory tests, in order of highest uptake.

Among ANC-W, HIV serology results were most frequently available (82%), followed by albuminuria (63%), while Hemoglobin results were least available (48%), as shown in Fig 1. Considering the cascade from test request to availability of result, the most frequent bottleneck for ANC-W was non-execution of requested tests, which was observed in 13% of women for HIV serology, in 23% for albuminuria and between 36–41% for the other five tests. The lack of a test request by ANC staff was second, but only for Hemoglobin and albuminuria (13% of women) and was <5% for the other 5 tests. Unavailability of results for executed test occurred for only 1–2% of the ANC-W. LAB-W most commonly had 5 (30%) or 4 (26%) test results (Table 6), in line with the expectation that LAB-W had already received HIV, and/or PU rapid testing at the level of the health posts. The lab requests of LAB-W most commonly included Syphilis (93%), followed by Blood group/Rhesus and Emmel test (83%), and Hemoglobin (65%). Of Hemoglobin requests 11% were not executed, but otherwise non-execution or non-availability of results was rare among LAB-W with a test request. The prior status of test results with lifelong validity (blood group/Rhesus factor and Emmel test) was documented for 7% of ANC-W and LAB-W, almost exclusively women with previous pregnancies.

**Table 6:** Adequacy of clinical management for abnormal test results (i.e. a result out of range or otherwise indicative of further clinical action).

	ANC-Women											LAB-Women		
Clinical Management adequate?	Result available	Abnormal results		Clinical management as recorded on card								Result available	Abnormal results	
				Not appropriate		Partly appropriate		Appropriate		N.A.				
	n	n	*%*†	n	%‡	n	%	n	%	n	%	n	n	%
Hemoglobin* (n,%)	597	308	*52%*	26	*8%*	229	*74%*	53	*17%*	0	*0%*	260	130	*50%*
Albuminuria (n,%)	780	28	*4%*	18	*64%*	5	*18%*	1	*4%*	4	*14%*	51	4	*8%*
HIV (n,%)	1.016	10	*1%*	1	*10%*	2	*20%*	5	*50%*	2	*20%*	127	1	*1%*
Syphilis (n,%)	741	6	*1%*	3	*50%*	1	*17%*	1	*17%*	1	*17%*	412	6	*1%*
Glycemia (n,%)	689	95	*14%*	72	*76%*	1	*1%*	3	*3%*	19	*20%*	318	38	*12%*
Emmel test (n,%)	696	41	*6%*	17	*41%*	4	*10%*	14	*34%*	6	*15%*	369	26	*7%*
Rhesus (blood group) (n,%)	728	37	*5%*	12	*32%*	0	*0%*	20	*54%*	5	*14%*	374	18	*5%*
**Total abnormal results (n,%)**	** **	**525**	** **	**149**	*28%*	**242**	*46%*	**97**	*18%*	**37**	*7%*	* *	**223**	** **

*Of which 2 in ANC and 3 in LAB group had severe anaemia, all with management not ok.

†percentage of women with results available for the respective test.

‡percentage of women with abnormal results.

ANC-Women: Pregnant women in the study who attended the facility for their first ANC visit. LAB-Women: Pregnant women in the study who were referred to laboratory by outside providers. N.A. = information not available

Adequate clinical management was recorded for 20/37 ANC-W with Rhesus-negative status (54%), and 5/10 for a HIV-positive status (Table 6). For other tests the appropriateness of recorded clinical management was lower. Overall, 97(18%) of 525 abnormal test results had a record of adequate management. The proportion of LAB-W with abnormal results was similar for each test as observed for ANC-W.

### Uptake patterns and turn-around times

**Fig 2:**
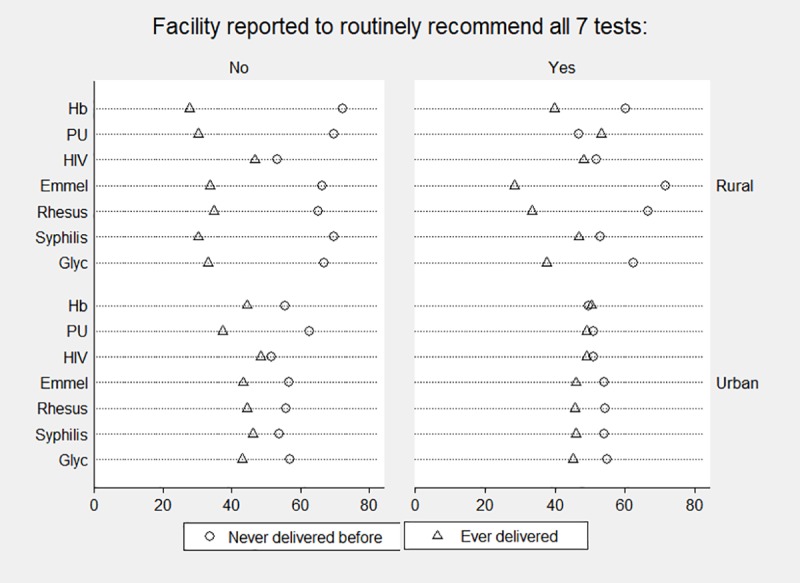
**Percentage ANC women with results for each recommended test, separate for those who never (circle) vs. ever delivered (triangle), stratified by facilities in rural vs. urban areas and whether the facility reported to routinely recommended all seven tests or not.** Number of rural facilities without routine recommendation of the 7 tests n = 2. Number of rural facilities with routine recommendation of the 7 tests n = 2. Number of urban facilities without routine recommendation of the 7 tests n = 3. Number of urban facilities with routine recommendation of the 7 tests n = 9.

Complete test uptake among ANC-W was lowest at the rural health facilities, 0–1% in three, and 24% in the fourth facility. When further stratified by test, parity, and routine recommendation all 7 tests, Hemoglobin testing was frequently left out at the rural facilities (Fig 2). Among women with prior deliveries, uptake of most tests was ±20–30%, except for HIV serology–and to some extent syphilis and PU—which had higher uptake. In urban health facilities that reported that all seven tests were routinely recommended, the uptake was highest and quite similar among women who ever vs. never delivered. Uptake was more equal for all tests, although HIV and PU still stood out. In the urban facilities without routine recommendation, uptake across tests and women with and without prior delivery experience was also quite similar, except for PU which was available for a quarter only.

**Fig 3:**
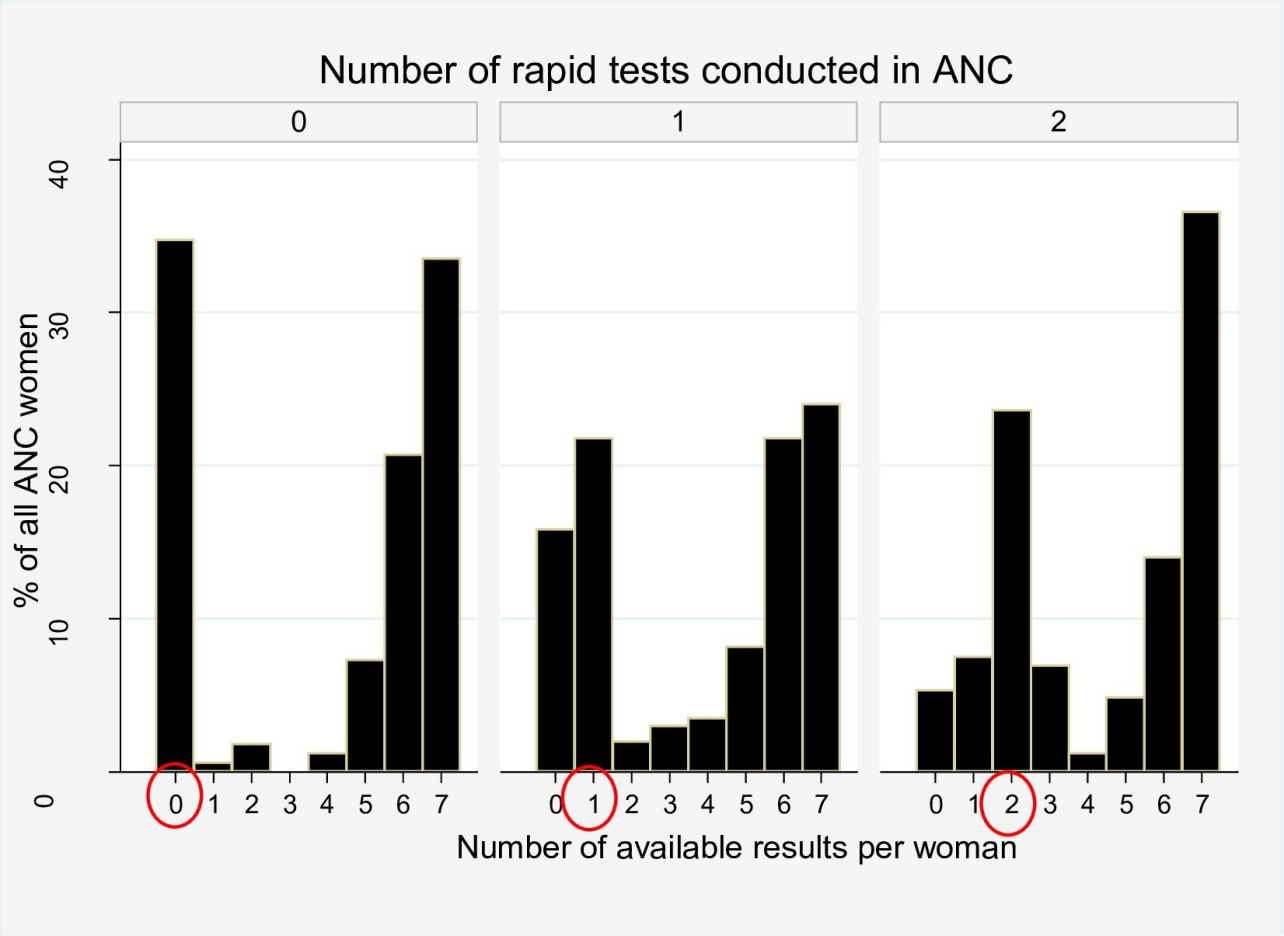
Distribution of the number of results available of the seven recommended tests per women attending ANC, stratified by the number of rapid tests conducted at ANC.

The complexity a woman encounters in getting her seven tests executed and obtain results depends on the number of rapid tests that were conducted in ANC vs. at the laboratory. As explained in Table 3, at facilities where HIV rapid testing is conducted at ANC, PU rapid testing at the laboratory on the same day, and other tests at the laboratory but not same-day, women require three moments at two places, while conducting all tests at the lab requires two moments at one place. For women with incomplete uptake, the number of available results peaked at the number of rapid tests that were available at ANC (Fig 3), but this was not associated with uptake in regression analysis.

For executed tests, test result availability for ANC-W was same-day for 83% and next day for 10%. For LAB-W this was 73% and 15% respectively. Test request, execution and clinical management for ANC-W with incomplete uptake was same day for 49%, and within one week for 75%. This was 21% same-day and 61% within one week for ANC-W with complete uptake of 7 results.

## Discussion

This analysis showed that among women attending for their first ANC visit at health facilities with laboratory capacity, uptake of the seven recommended laboratory tests [15] was low: only one in three obtained the complete set of test results. However, uptake varied widely between facilities, was lower in rural facilities, and more so among women with earlier deliveries. In urban facilities uptake was higher and depended less on women’s prior delivery status, especially in facilities that routinely recommended all seven tests. Failure to execute a requested test was the most common bottleneck, while, once executed, missing tests results were uncommon (<2%).

Low uptake of ANC tests despite available laboratory infrastructures and commodities, and high (first) ANC attendance indicate missed opportunities for quality ANC in Senegal. Even more so for rural women who, in west-Africa, are already far less likely to receive ANC than their urban counterparts [8].

The results of the anthropological components of the mixed-methods SociaLab project (published elsewhere [10]) give us insights that helped us understand the results of the quantitative analyses presented in the current manuscript. For pregnant women, reasons for non-execution of requested ANC laboratory tests mostly related to difficulties in obtaining money to pay for the tests and for the multiple travels for clinic and laboratory visits. In Senegal women do not have to pay for delivery care in public facilities [19], but payment is required for ANC consultation, registration, medicines, laboratory diagnostic tests -which are expensive when considering poverty-, and echography [10]. Illiteracy, which is common among women and men [14], aggravated the financial barrier as not being able to read the test request and thus not knowing how much to request or precisely what to request money for made it hard for women to raise sufficient money, usually from their husbands, for tests and for transportation to collect results.

The anthropological study also revealed the role of midwives as gatekeepers. At the health facilities midwives reported anticipating the financial hardship of women. They tried to limit the prescription of medicines and requests for test that women could not afford, by prioritizing tests that they judged most important. Or they skipped the most expensive ones (e.g. Hb) and instead relied on clinical symptoms, a common aspect of clinical medicine in SSA [1, 4, 20]. In addition, midwives faced heavy workload, shortage of furniture, equipment and consumables in most facilities, and poor pay. A variety of reasons given by midwives for not explaining to women about the tests were: lack of time, forgetting (due to high workload), illiterate women not being able to understand, anticipation that women might refuse the test (for HIV), a language barrier, the lack of clear national guidelines for ANC screening test creating inconsistency in which tests were recommended, not filling a request form for tests that were faulty at the facility’s own lab, or in anticipation of stock-outs. At the laboratory level reported barriers for test execution were the cost of the test, stock-outs of reagents, and broken equipment.

Lack of confidence in laboratory results for clinical management due to lack of equipment, inadequate supply-chain management for consumables and reagents, poor equipment maintenance, lack of clear policies, and insufficient leadership, points out the importance of laboratory capacity strengthening efforts [2]. And the need for human resource management interventions, which can improve health workers' performance, taking into account that different contexts produce different outcomes [21]. During a result dissemination workshop the study findings were shared, barriers were discussed among different stakeholders [22], and an implementation plan was developed.

Whereas a more in-depth understanding through triangulation of data from different research methods was a strength of our study, it also pointed to limitations. Among pregnant women approached for anthropological interviews in the community, verification of ANC cards suggested that ANC test utilization was even lower than among the women we report on in this manuscript, especially among women attending ANC at health posts, and tests were more often not requested [10]. The differences may be due to sampling or incompleteness of ANC cards, but study procedures likely contributed: in each facility a midwife was trained to recruit and attend to study participants and record clinical data on specially designed study ANC cards to facilitate data entry. This because in routine care standard antenatal cards were often not completed fully, a problem that is more common in this type of studies [23]. The study midwives probably became more mindful of requesting the test, which were then not followed on by the women because of financial constraint, thus suggesting a different bottleneck in the cascade, and possibly a ‘study effect’ towards more complete test request. Equally, higher availability of test results may have occurred from laboratory staff more mindful of delivering better quality of test is results in the context of this study. Collecting data in 16 different facilities was intended to capture variation. Even though the results reported may represent a more optimistic overview of the ANC cascade compared to facilities not participating to the study, the gaps we described remain substantial.

Other limitations apply to the multilevel regression analysis, which was explorative. The available women characteristics were limited to information routinely collected during ANC, and did not include additional assessment of women’s individual socioeconomic status, literacy level or distance to the facility. With 16 facilities power was low and failure to demonstrate an effect of facility level characteristics should not be interpreted as if these aspects are irrelevant. Also, facility assessments were cross-sectionally, so e.g. stock-outs or breakdowns may not have coincided with the full enrolment period. The analysis however suggests that most variation in uptake was explained at the facility level, which invites for interventions targeting the health facility and management level. Moreover, it tones down the blame that health workers tend to put on the women for low uptake of tests. Facilities that routinely recommended all tests had higher uptake of the complete set underscoring the importance of guideline implementation. Longer opening hours of the lab was inversely associated with uptake, which is counterintuitive, as short opening hours of the lab had been suggested as a reason for low uptake by ANC staff. It may be an indication of another unmeasured facility characteristic, but may also indicate that intuitive solutions (i.e. increasing opening hours) may improve client-friendliness, but on its own may not necessarily solve the uptake problem. The national recommendation changed from 6 to 7 tests (glycaemia was added) during the study period [15], but this had little impact since all facilities already considered glycaemia testing as part of the package at study onset and glycaemia uptake was quite similar to e.g. syphilis testing. A strength of our study is the detail in uptake of different tests, compared to the Senegalese Demographic and Household Surveys (DHS) that measured uptake of urine and blood sampling (90% for urine- and 84% for blood sampling among women attending ANC in 2016) without specifying whether and which tests were carried out on the specimens [24].

Low uptake of ANC tests is not unique to the setting of our study, and was even lower in a study in Mozambique [23] that aimed at improving uptake of antenatal syphilis screening through integration of HIV and syphilis rapid tests in ANC. In our study HIV rapid testing, which is managed vertically by the Senegalese HIV program, had the highest uptake, and confirms the perception that point-of-care (POCT) technologies increase access to health services. However, when rapid tests for HIV (free) and/or proteinuria (not free) were conducted at ANC, a subgroup of women had results for these tests only. Similarly, experiences with POCT for syphilis [25] and CD4+ T-cell enumeration [26] showed wide variability in uptake, indicating the potential for detrimental effects in some settings or sub-populations. If importance of a full ANC laboratory tests packages could be promoted through a health systems approach, then goals and funding of vertical programs could be utilized to improve lab test uptake and quality and levels of service delivery. A similar approach is used in other laboratory capacity building initiatives, were support for vertical disease programs is combined with horizontal health system approaches [2, 4], and vertical program monitoring could be a monitoring strategy [27].

In conclusion, our study showed that uptake of recommended laboratory tests among first ANC attendants was low, and lowest in rural facilities and among women with earlier deliveries, illustrating challenges to test uptake even when laboratory testing capacity is in place. Large differences between facilities underscore the importance of management, policy, and the importance of considering local context in order to improve service delivery to expectant mothers by identification of specific issues at each facility that require intervention.

## Supporting information

S1 Supporting File Fiche de suivi / ANC card. (PDF)

S2 Supporting File Facility questionnaires. (PDF)

S1 Dataset (XLS)
