# Supplementary material for: A study to better understand under-utilization of laboratory tests for antenatal care in Senegal
Source: PLoS One. 2020 Jan 9;15(1):e0225710. doi: 10.1371/journal.pone.0225710 (PMC6952088; doi:10.1371/journal.pone.0225710)
Supplement: S1 Supporting File — (PDF) [file pone.0225710.s001.pdf]

Nom Structure :______________________________ Code Structure :______________ N°Identification :______________ Réf : EN-11 /02/DL - DA : 11.07.2013 – V2

Lieu CPN : □ dans la structure ; □ en dehors de la structure : *□PS ;□CS ; □Privé* - Age de la grossesse : □1er trim ; □2ème trim ; □3ème trim -Age gestante : _____ans - Gestité : Parité:

CPN: DEMANDE DE TESTS	LABORATOIRE : TESTS DEMANDES	LABORATOIRE : RESULTATS	CPN : PRISE EN CHARGE
Date : __ /__ / ______	Date : __ /__ / ______	Date : __ /__ / ______	Date : __ /__ / ______
**PRESCRIPTEUR**<br>Prénoms : __________<br>NOM : ____________<br><br>Qualification : □SF □GO □Inf<br>□Autre, à spécifier<br>______________	**RESPONSABLE LABORATOIRE**<br>Prénoms : ______________<br>NOM : __________________<br><br>Qualification : □TE □TS □BIO<br>□Autre, à spécifier<br>______________________	**RESPONSABLE LABORATOIRE**<br>Prénoms : ______________________<br>NOM : __________________________<br><br>Qualification : □ TE □TS □BIO<br>□Autre, à spécifier<br>____________________________	**PRESCRIPTEUR**<br><br>Prénoms : ____________________ NOM : _______________<br><br>Qualification : □SF □GO □Inf<br>□Autre, spécifier<br>____
□ GS/ RH	□ GS/ RH	□A+ □B+ □AB+ □O+ □A- □B- □AB- □O-<br>DU □ Nég □ Pos	
□ Test d'Emmel	□ Test d'Emmel	□ Nég □ Pos	
□ Alb/Sucre	□ Alb/Sucre	□ Nég □ Pos	
□ Glycémie	□ Glycémie	Glycémie : ________________ g/l	
□ B-Test	□ B-Test	RPR : □ Nég □ Pos ; TPHA : □1/16 ; □1/64 ;□1/256	
□ A-Test	□ A-Test	□ Nég ; □ Pos : □A1 ; □A2 ; □A1+2	
□ NFS /Hg	□ NFS /Hg	Taux Hb : ________ mmol/L ou _______ g/dL	
□ Créatinine	□ Créatinine	Taux Créat : ______1 mg/dl ou ______µmol/l	
□ Acide urique	□ Acide urique	AU : __________ mg/L ou __________ µmol/L	
□ AgHBs	□ AgHBs	□ Nég □ Pos	
□ Prélèvement Vaginal : Recherche de germes :	□ Prélèvement Vaginal : Recherche de germes :	*Streptococcus agalactiae* □ Nég □ Pos	
		Escherichia coli □ Nég □ Pos	
		Trichomonas vaginalis □ Nég □ Pos	
		Candidose vaginale □ Nég □ Pos	
		Neisseria gonorrhoeae □ Nég □ Pos	
		Vaginose bactérienne à *Gardnerella vaginalis*<br>et /ou *Mobiluncus spp* □ Nég □ Pos	
□ Mycoplasmes	□ Mycoplasmes	*Ureaplasma urealyticum* : □ Nég ;□ Pos (> 10^4 Ucc/ml)<br>*Mycoplasma hominis* : □ Nég ; □ Pos (> 10^4 Ucc/ml)	
□ *Chlamydia trachomatis*	□ *Chlamydia trachomatis*	□ Nég □ Pos	
□ Sérologie Toxoplasmose	□ Sérologie Toxoplasmose	IgG : _________ UI/ml ; IgM : Titre ____________	
□ Sérologie Rubéole	□ Sérologie Rubéole	IgG :__________UI/ml ; IgM : Titre ____________	
