# Supplementary material for: A study to better understand under-utilization of laboratory tests for antenatal care in Senegal
Source: PLoS One. 2020 Jan 9;15(1):e0225710. doi: 10.1371/journal.pone.0225710 (PMC6952088; doi:10.1371/journal.pone.0225710)
Supplement: S2 Supporting File — (PDF) [file pone.0225710.s002.pdf]

	ENREGISTREMENT	Réf : EN-09 /02/DL	VERSION 03	
	Grille d'Evaluation des Consultations Prénatales	Date d'application : 12.07.2013		
		Page1.34		

Etude des facteurs socio-culturels et historiques limitant le recours au laboratoire dans les soins prénataux au Sénégal, Mali et Burkina Faso

Cet outil permettra d'évaluer le niveau de connaissance des prestataires par rapport aux tests prénataux

IIème PARTIE

Nom de la structure :……………………………………………………
Type de structure : ☐ Centre de santé ☐ EPS1 ☐ Clinique privée
Localisation : ☐ Rurale ☐Urbaine
Durée de l'enquête : du ___________ /_________/ au ___________ /__________/______________
Prénoms et nom de l'enquêteur : ……………………………………………
Signature :

Prénoms et nom de l'enquêté : ……………………………………………….

Tel : ……………………………….. Mail : …………………………..

Qualification :

☐Sage femme ☐ Maitresse sage femme ; ☐Infirmière

Signature :

V .CONNAISSANCES DES TESTS RECOMMANDES PAR LA SR A CHAQUE CPN

Q49. Connaissez-vous sont les tests préconisés lors de la CPN1 par la SR ? ☐ Oui ☐non

Q50.Si oui, dites lesquels ?

**Tests Obligatoires (TO)**	**Tests conseillés (TC)**	**Tests (TOF)**
☐ Groupage sanguin<br>☐ Recherche d'agglutinines irrégulières si Rhésus négatif (Coombs indirect)<br>☐ NFS<br>☐ Test d'Emmel	☐ AgHBS<br>☐ Ac Anti HCV<br>☐ Frottis cervicovaginal	☐ Sérologie toxoplasmose<br>☐ Sérologie Rubéole<br>☐ Glycémie à jeun

□ Sérologie syphilitique (RPR/TPHA)<br>□ Sérologie HIV (*counceling* dépistage à l'initiative du prestataire)<br>□ Recherche d'albumine avec les bandelettes. A partir de deux croix, demander une albuminurie des 24 h<br>□ Recherche d'une maladie rénale chronique par les bandelettes urinaires multifonction et le dosage des la créatinémie		*Examens orientés en fonction du contexte* :<br>□Nitrites ; □Glycosurie ; □Leucocyturie sur bandelettes ;<br>□Protéinurie des 24 h ;□ECBU ; □PV ; □Créatininémie ;<br>□Calcémie ;□Protidémie ;□Albuminémie ;□Uricémie ;<br>□Transaminases ; □Taux de prothrombine ;□Dosage CD4

Q51. Connaissez-vous sont les tests préconisés lors de la CPN2 par la SR ?

□ Oui □Non

Q52.Si oui dites lesquels ?

**Tests Obligatoires (TO)**	**Tests conseillés (TC)**	**Tests (TOF)**
□ RPR/TPHA si test négatif au 1er trimestre<br>□ Albumine urinaire (bandelettes)<br>□ Albumine des 24 h à partir de 2 croix<br>□ Créatinémie à partir de 2 croix	□ AgHBs au 6ème mois<br>□ Ac Anti HCV	□ NFS si anémie clinique<br>□ Test de O'Sullivan au 6ème mois si antécédents de macrosomie fœtal<br>□ PV

Q53. Connaissez-vous sont les tests préconisés lors de la CPN3 par la SR ? ☐ Oui ☐ Non

Q54.Si oui dites lesquels ?

**Tests Obligatoires (TO)**	**Tests conseillés (TC)**	**Tests (TOF)**
☐ RPR/TPHA si test négatif au 1er trimestre<br>☐ Albumine urinaire (bandelettes)<br>☐ Albumine des 24 h à partir de 2 croix<br>☐ Créatinémie à partir de 2 croix<br>☐ Sérologie rétrovirale HIV		☐ NFS si anémie clinique<br>☐ PV/examen bactériologique

Q55. Connaissez-vous sont les tests préconisés lors de la CPN4 par la SR ? ☐ Oui ☐Non

Q56.Si oui dites lesquels ?

**Tests Obligatoires (TO)**	**Tests conseillés (TC)**	**Tests (TOF)**
☐ RPR/TPHA si test négatif au 1er trimestre<br>☐ Albumine urinaire (bandelettes)<br>☐ Albuminine des 24 h à partir de		☐ NFS si anémie clinique<br>☐ PV/examen bactériologique

2 croix<br><br>☐ Créatinémie à partir de 2 croix<br>☐ Sérologie HIV		

VI CONNAISSANCE DES TESTS DE DIAGNOSTIC DE LABORATOIRE ET DE LA CONDUITE A TENIR FACE A UNE PATHOLOGIE DE LA MERE

Q57.Est ce que les prestataires ont une bonne maitrise des tests et la conduite à tenir en cas de résultats anormaux

**TESTS**	**JUSTIFICATIFS**	**PRISE EN CHARGE DE RESULTATS ANORMAUX**
**1.GS/ RH**	➢ **Indications**<br>☐ Recherche de groupe sanguin et facteur Rhésus<br>☐ Pour une éventuelle transfusion<br>➢ **Incidences chez la maman**<br>☐ Connaissance du groupe sanguin et facteur Rhésus<br>☐NSP ; ☐ RAS ; ☐Autre, à spécifier : ……………<br><br>➢ **Incidences chez l'enfant**	☐ Recherches d'agglutininines irrégulières (RAI) en cas de rhésus négatif de la mère<br>☐ Sérothérapie/vaccination avec le sérum anti D en cas d'incompatibilité de rhésus dans les 72h après l'accouchement de la maman<br>☐NSP ; ☐ RAS ; ☐ Autre, à spécifier :<br>……………………………………..

	☐ Incompatibilité de rhésus entre mère-enfant<br>☐ Hémolyse<br>☐ Mort né<br>☐NSP ; ☐ RAS ; ☐Autre, à spécifier : ……………	

**2.Test d'Emmel**	➢ **Indications**<br>□ Dépistage de la Drépanocytose<br>➢ **Incidences chez la maman**<br>□ Anémie Sévère<br>□ Evènements vaso-occlusifs (crises, syndrome thoracique, toxémie gravidique)<br>□ Infections urinaires<br>□ Aggravations de complications préexistantes (cardiaque, rénal, pulmonaire etc)<br>□NSP ; □ RAS ; □ Autre, à spécifier ……………<br><br>➢ **Incidences chez l'enfant**<br>□ Risques plus élevés de complications fœtales (Fausses couches, retard de croissance avec hypotrophie, prématurité…)<br>□ Enfant drépanocytaire<br>□NSP ; □RAS ; □Autre, à spécifier : …………	□ Electrophorèse de l'Hb pour le typage;<br>□ Antalgiques ; □ Myorelaxants en présence de symptômes (Douleurs articulaires, fatigue intense, etc<br>□Repos ; □Oxygénation ; □Hydratation ; □Réchauffement<br>□RV rapprochés ; □hospitalisation<br>□ Transfusion répétitive de petites quantités de sang frais,<br>□ ECBU<br>□ Prélèvements vaginaux<br>□Acide folique 1 à 5 mg/j<br>□NSP ; □ RAS ; □ Autre, à spécifier :<br>………………………………

**3.Alb/Sucre**	**Glycosurie (glucose dans l'urine)**<br>➢ **Indications**<br>☐ Diabète gestationnel<br>➢ **Incidences chez la maman**<br>☐ Accouchement prématuré<br>☐ Césarienne le plus souvent<br>☐ NSP ; ☐ RAS ; ☐ Autre, à spécifier : ………..<br>➢ **Incidences chez l'enfant**<br>☐ Macrosomie fœtale<br>☐ Hypoglycémie à la naissance<br>☐ Défaut de maturation pulmonaire<br>☐NSP ; ☐ RAS ; ☐ Autre, à spécifier : …………..	☐Corrélation résultats avec Clinique;<br>☐Conseils hygiéno-diététiques<br>☐Insuline<br>☐Confirmation avec la glycémie à jeun<br>☐Référence en diabétologie<br>☐Référence chez le Gynécologue-Obstétricien<br>☐NSP ; ☐ RAS ; ☐ Autre, à spécifier :<br>……………………………

	**Albuminurie (Albumine dans l'urine)**<br>➢ **Indications**<br>☐ Recherche d'albumine dans l'urine<br>➢ **Incidences chez la maman**<br>☐ Toxémie gravidique : protéinurie + hypertension artérielle +oedèmes<br>☐ Préclampsie **:** tension artérielle très élevée +convulsions+Oedèmes<br>☐ Hématome rétroplacentaire (HRP)<br>☐ Décès maternel<br>☐ NSP ; ☐RAS ; ☐ Autre, à spécifier : ....<br>➢ **Incidences chez l'enfant**<br>☐ Hypotrophie<br>☐ Hématome rétroplacentaire (HRP)<br>☐ Souffrance fœtale<br>☐ Mort fœtale<br>☐ ☐NSP ; ☐ RAS ; ☐ Autre, à spécifier :	☐ Antihypertenseurs<br>☐ Repos le plus souvent lors d'une hospitalisation.<br>☐ Extraction en urgence de l'enfant par césarienne en cas absence d'amélioration ou d'aggravation du tableau clinique<br>☐ Conseils hygiéno-diététiques<br>☐NSP ; ☐ RAS ; ☐ Autre, à spécifier :<br>................................

**4.BW**	➢ **Indications**<br>☐ Diagnostic de la Syphilis<br>☐ Infection sexuellement transmissible<br>➢ **Incidences chez la maman**<br>☐ Avortement spontané<br>☐NSP ; ☐ RAS ; ☐ Autre, à spécifier : …………<br><br>➢ **Incidences chez l'enfant**<br>☐ Mort fœtal intra utérin (MFIU)<br>☐ Syndrome de syphilis congénital<br><u>Signes précoces</u> : ☐Hépato splénomégalie ; ☐ Ictère cutanéo-muqueux<br><u>Signes tardifs</u> : ☐Anomalies dentaires, ☐Surdité, ☐Atteintes neurologiques, ☐Osseuses<br>☐NSP ; ☐RAS ; ☐Autre, à spécifier…………………	➢ **Chez la maman**<br>☐ Benzathine pénicilline G 2,4 MUI en une injection IM /sem /3sem. (syphilis tardive) **(PNP-SR)**<br>☐ Benzathine pénicilline G 2,4 MUI en IM (syphilis I^{aire}, 2nd^{aire} ou latente précoce) **(OMS et CDC)**<br>☐ Benzathine pénicilline G 2,4 MUI en IM, 3xsem (syphilis latente tardive ou une syphilis latente dont on ne connaît pas la durée) **(OMS et CDC)**<br>☐ Erythromycine 500 mgx4/j/14jrs chez la maman (syphilis tardive) **(PNP-SR)**<br>☐ En cas de neurosyphilis, référence en neurologie<br>☐ Traitement partenaire<br>➢ **Chez le nouveau-né infecté ou fortement infecté**<br>☐ Benzathine pénicilline 100 000 à 150 000 UI/kg/j /10j **(OMS et CDC)**<br>➢ **Chez le nouveau-né asymptomatique d'une mère séropositive**<br>☐ Benzathine pénicilline 50 000 UI/kg/j /, dose unique **(OMS et CDC)**

		☐ RAS ; ☐NSP ; ☐ Autre, à spécifier<br>………………………….….
**5. Sérologie HIV**	➢ **Indications**<br>☐ Dépistage de l'infection à VIH<br>➢ **Incidences chez la maman**<br>☐ Infections opportunistes liées au VIH<br>☐NSP ; ☐ RAS ; ☐ Autre, à spécifier : ……… …<br>➢ **Incidences chez l'enfant**<br>☐Prévention Transmission Mère Enfant (PTME)<br>☐ Manifestations cliniques liées au VIH : retard pondéral, troubles de la croissance, dermatologiques, cardiaques, etc ;<br>☐NSP ; ☐ RAS ; ☐ Autre, à spécifier : ……………	☐ Référence au Laboratoire pour confirmation et typage<br>☐ Référence au Laboratoire pour dosage CD4<br>☐ Traitement antirétroviral (ARV) pour la maman dès l'annonce du résultat<br>☐ ARV pédiatriques dès la naissance (NVP)<br>☐ Référence pour PEC si confirmation faite (Recommandations programme national)<br>☐NSP ; ☐ RAS ; ☐ Autre, à spécifier :<br>……………………
**6. Taux d'Hémoglobine**	➢ **Indications**<br>☐ Diagnostic biologique de l'Anémie ferriprive<br>☐NSP ; ☐ RAS ; ☐ Autre, à spécifier : ……………	**PEC en fonction du type d'anémie (PNP-SR)**<br>☐**Anémie sévère** : ☐ Hg < 7 g/dl ; ☐ Transfusion et exploration ;

	➢ **Incidences chez la maman**<br>☐ Anémie ferriprive gravidique<br>☐ Fatigue ; ☐Céphalées ; ☐Vertiges **;** ☐Tachycardie<br>☐ RAS ; NSP ; Autre, à spécifier…………………<br><br>➢ **Incidences chez l'enfant**<br>☐ Hypotrophie fœtal<br>☐ Mort fœtale in utero<br>☐ Prématurité de l'enfant<br>☐ Infections néonatales<br>☐ Méningites<br>☐ Détresse respiratoire<br>☐ RAS ; NSP ; Autre, à spécifier…………………	☐**Anémie modérée** : Hg entre 7 et 11 g/dl ; ☐ 120mg fer et 400 µg acide folique /j/6 mois ; ☐ Vitamine C ; ☐ Référer si absence d'amélioration au bout d'un mois, pour exploration avec NFS, fer sérique<br>☐ Conseils hygiéno-diététiques ;<br>☐ RAS ; ☐NSP ; ☐ Autre, à spécifier :<br>…………………………

CONCLUSION

- Q59. Quelles appréciations faites-vous de vos CPN?

**Points forts**	
**Points faibles**	
**Recommandations**	

- Q60.Quelles appréciations faites-vous des prestations du laboratoire ?

**Points forts**	
**Points faibles**	
**Recommandations**	

Etude des facteurs socio-culturels et historiques limitant le recours au laboratoire dans les soins prénataux au Sénégal, Mali et Burkina Faso

Cet outil permettra d'évaluer :

- *La demande et l'utilisation des tests prénataux dans la prise en charge des femmes enceintes*
- *Le fossé entre les recommandations et la pratique de la SR*
- *Les barrières liées à l'utilisation des tests*

1ERE PARTIE

Nom de la structure : ……………………………………………

Type de structure : ☐ Centre de santé ☐ EPS1 ☐ Clinique privée

Localisation : ☐Rurale ☐Urbaine

Durée de l'enquête :

Prénoms et nom de l'enquêteur :

Signature :

Prénoms et nom de l'enquêté :

Tel __________________________; Mail :

Qualification :

☐Sage femme ; ☐Maitresse sage femme ; ☐Infirmière

Signature :

I.ORGANISATION DE LA CPN

I.1 RESSOURCES HUMAINES

Q1.Quel est le nombre de prestataires en service à la CPN ?

Prestataires	Nombre		
	Homme	Femme	Total
1. Gynécologue			
2. Sage-femme			
3. Infirmiers			
4. Aide infirmier			
5. Assistant social			
6. Autres, à spécifier …………………..			
Total			

I.2 LES OUTILS DE LA CPN

Q2.Cochez les outils disponibles

- ☐ Registre CPN du Programme National de la Santé de la Reproduction
- ☐ Agenda de Rendez-vous
- ☐ Registre de consultation générale
- ☐ Autres, spécifier ____

I.2.1 LES REGISTRES

Q3.Est-ce que toutes les rubriques du registre de CPN doivent être remplies?

☐OUI ☐ NON

Q4.Cochez les tests pour lesquels les résultats sont renseignés systématiquement dans le registre de CPN ?

TESTS	
☐ Groupage Sanguin/Rhésus	Liste minimale / Liste maximale
☐ Test d'Emmel	
☐ Sérologie syphilitique	
☐ Albumine/Sucre	
☐ Glycémie	
☐ Sérologie HIV	
☐ NFS -Taux d 'hémoglobine	
☐ AcAntiHCV	
☐ AgHbs	
☐ Sérologie toxoplasmose	
☐ Sérologie rubéole	
☐ Agglutinines irrégulières	
☐ Prélèvement Vaginal	
☐ Recherche directe de *Chlamydia trachomatis*	
☐ Recherche de Mycoplasmes	

I.2.2 CARNET DE CPN

Q5.Combien coute un carnet individuel de santé ☐ ………………CFA ; ☐ Gratuit

Q 6. Est-ce que la possession d'un carnet individuel de santé est une obligation?

Oui ☐ Non ☐

Q 7. En 2012, y' a-t-il eu rupture de stock de carnet de CPN ? Oui ☐ Non ☐

(si non passez à la question suivante)

Q8.Si oui, pour combien de temps ? (mois, jours..) ……………..

Q9.Qui remplit les carnets ?

- ☐ La maîtresse sage femme
- ☐ Les sages-femmes
- ☐ Le gynécologue-obstétricien
- ☐ L'aide infirmière
- ☐ Tout prestataire ayant consulté
- ☐ Autre, à spécifier : ……………………………….

Q10. Les résultats des analyses doivent-ils être renseignés dans le carnet ?

Oui ☐ Non ☐

Q11. Quels sont les résultats consignés en général dans les carnets?

TESTS		
☐ Groupage Sanguin/Rhésus	Liste minimale	Liste maximale
☐ Test d'Emmel		
☐ Sérologie syphilitique		
☐ Albumine/Sucre		
☐ Glycémie		
☐ Sérologie HIV		
☐ NFS		
☐ AcAntiHCV		
☐ AgHbs		
☐ Sérologie toxoplasmose		
☐ Sérologie rubéole		
☐ Agglutinines irrégulières		
☐ Prélèvement Vaginal		
☐ Recherche directe de *Chlamydia*		

trachomatis
☐ Recherche de Mycoplasmes

Q12.Pourquoi <u>tous</u> les résultats ne figurent pas dans les carnets ?

- ☐ Temps
- ☐ Place
- ☐ Pas recommandé (confidentialité ex HIV)
- ☐ Oublis
- ☐ Tests non réalisées
- ☐ Absence d'espace prévue pour les autres tests
- ☐ Autre, à spécifier ………………………………..

Q13.Existe –t-il d'autres outils dans lesquels sont consignés les résultats <u>individuels</u> des femmes ? Si oui, les lister :

………………………… … …………………. ………………………

Q14.Les prises en charge/interventions <u>doivent</u>-elles être renseignées dans le carnet ?

Oui ☐ Non ☐

Q15.Si non, ou doivent-elles être consignées ? ……… …………….

I.3 LOGISTIQUE

Q16. Existe t-il suffisamment de chaises/bancs (pour les femmes et accompagnatrices (eurs) ?

(A évaluer durant tout le séjour dans la structure)

☐ Oui ☐ Non

Q17. Est-ce qu'il existe des toilettes <u>accessibles</u> aux femmes enceintes ?

☐Oui ☐Non

Q18. Appréciez la propreté des toilettes.

☐ Très satisfaisante ☐ Satisfaisante ☐ Moyen ☐ Peu satisfaisante

Q19. Existe t-il des animations/distractions pendant l'attente ? Cochez si disponible

- ☐ TV
- ☐ Education sanitaire
- ☐ Autre, à spécifier : ………………………………………

Q20. Appréciez la propreté de la salle d'attente (*Toute la journée*)

☐Très satisfaisante ☐ Satisfaisante ☐Moyen ☐ Peu satisfaisante

Q21. Quel est le type de climatisation dans la salle d'attente ?

- ☐ Brasseur d'air
- ☐ Climatiseur
- ☐ Brasseur d'air et climatiseur
- ☐ Aucun

Q22. Quel est le temps d'attente pour la CPN ? (*A estimer en minutes sur un échantillon de 10 femmes)*

	n° 1	n°2	n°3	n°4	n°5	n°6	n°7	n°8	n°9	n°10
Heure d'Arrivée T1										
Heure de Sortie T2										
Temps d'attente (T2-T1)										
……… min										

II. NORMES ET SYSTEMES EN PLACE POUR LA CPN

Q23. Les recommandations nationales en terme de Santé de la Reproduction et soins prénataux sont elles-connues et mises en place dans les services ? *(Vérifier la disponibilité des doc. Politiques, Normes et Protocoles)*

☐ Oui ☐ Non

Si oui, lister les documents existants ; Si non passez à la question Q26

……………………………………… ………………………………………………

Q24. Est-ce qu'il existe des procédures (SOP) décrivant de manière claire le déroulement et le contenu de la CPN dans le service ? *(vérifier)*

☐ Oui ☐Non

Si oui, lister les documents existants

……………………………… ……………………………………………………………

Q25.Si oui, sont-elles accessibles aux prestataires? *(vérifier)*

☐ Oui ☐ Non

Q26. Connaissez-vous les recommandations nationales en termes de prescription du bilan pour les soins prénataux ?

☐ Oui ☐ Non

Si oui, lister les documents existants : ………………………………

Si non, passez à la question Q29

Q27.Est ce que vous les respectez pour prescrire le bilan ?

☐ Oui ☐ Non

Q28.Si non, quelle en est la raison ?

Q29. Tenez-vous compte des résultats des grossesses antérieures pour la demande de nouveaux tests ?

Oui☐ Non ☐

Q30. Quelles sont les analyses demandées aux primigestes ?

TESTS
☐ Groupage Sanguin/Rhésus
☐ Test d'Emmel
☐ Sérologie syphilitique
☐ Albumine/Sucre
☐ Glycémie
☐ Sérologie HIV
☐ NFS -Taux d 'hémoglobine
☐ AcAntiHCV
☐ Acide urique
☐ AgHbs
☐ Sérologie toxoplasmose (en cas d'avortement à répétitions)
☐ Sérologie rubéole (en cas d'avortement à répétitions)
☐ Agglutinines irrégulières
☐ Prélèvement Vaginal
☐ Recherche directe de *Chlamydia trachomatis*
☐ Recherche de Mycoplasmes

Liste minimale (Groupage Sanguin/Rhésus à NFS -Taux d 'hémoglobine)

Liste maximale (Groupage Sanguin/Rhésus à Recherche de Mycoplasmes)

Q31. Quelles sont les analyses demandées à celles qui sont à plus de leur 1ère grossesse ?

TESTS
☐ Groupage Sanguin/Rhésus
☐ Test d'Emmel
☐ Sérologie syphilitique
☐ Albumine/Sucre
☐ Glycémie
☐ Sérologie HIV
☐ NFS – taux d 'hémoglobine
☐ Acide urique
☐ AgHbs
☐ Sérologie toxoplasmose
☐ Sérologie rubéole
☐ Agglutinines irrégulières
☐ Prélèvement Vaginal
☐ Recherche directe de *Chlamydia trachomatis*
☐ Recherche de Mycoplasmes

Liste minimale (Groupage Sanguin/Rhésus – NFS – taux d 'hémoglobine)

Liste maximale (Groupage Sanguin/Rhésus – Recherche de Mycoplasmes)

Q32.Quels sont les *tests rapides* réalisés présentement par les sages femmes ?

☐ Albumine/Sucre ☐ HIV ☐TDR Paludisme ☐Aucun
☐ Autre, à spécifier ……………..

Q33.Quel est le délai de rendu des résultats des *tests rapides* effectués par les sages femmes ?

Tests	Durée phase pré-analytique (prélèvement et partie pratique) (min)	Durée phase analytique (réalisation du test (min.)	Durée phase post analytique (Validations enregistrements, rendu du résultat aux patients
Albumine/Sucre			
TDR Paludisme			

HIV			
Autre, à spécifier …………			

Q34. Qui réalise les *tests rapides* ?

- ☐ Le gynécologue
- ☐ La sage femme
- ☐ L'infirmier
- ☐ Autre, à spécifier ……………………………………………….

Q35.Qui est chargé de la validation technique des tests ?

- ☐ Le gynécologue
- ☐ La sage femme
- ☐ L'infirmier
- ☐ Autre, à spécifier ……………………………………………….

Q36.Au cas où les *tests rapides* sont positifs, quelle suite est donnée aux résultats ? Cochez la conduite à tenir

	Albuminurie	Glycosurie	HIV	TDR Paludisme	Autre
Un traitement est initié					
Le test est validé par un autre test/une autre méthode					
La référence chez le Gynécologue-Obstétricien pour une PEC					
La référence chez le Gynécologue-Obstétricien pour une PEC					
Rendez vous plus rapproché					
Autre, à spécifier ………………………………					

Q37. Listez les procédures (SOP) décrivant le type de PEC en cas de pathologies détectées ?

**Tests**	**SOPS**
Alb/Sucre	
HIV	
TDR Paludisme	
Autre, à spécifier	

III.DEROULEMENT DE LA CPN

Q38. Etes- vous tenue d'expliquer l'importance de la CPN aux femmes enceintes ?

☐ Oui ☐ Non

Q39. Si oui, êtes –vous tenue d'expliquer l'utilité des tests et l'implication des résultats sur la santé de la femme enceinte ?

☐ Oui ☐ Non

Q40.Qu'est ce qui est prévu lorsque la femme n'a pas respecté le rendez- vous ?

- ☐ On lui donne un autre rendez-vous
- ☐ On la prend en consultation, le même jour
- ☐ On la sensibilise
- ☐ Autre, à spécifier…………………………………………………………

Q41. Qu'est ce qui est prévu lorsque la femme revient sans les résultats du bilan biologique ?

- ☐ On ne la reçoit pas
- ☐ On se limite à l'examen clinique pour la prise en charge
- ☐ On la sensibilise
- ☐ Autre, à spécifier ………………………………………..

Q42.L'Examen clinique est-il réalisé à chaque CPN ?

☐ Oui ☐ Non

Q43.Cochez les paramètres mesurés aux différentes CPN ?

	Clinique				Biologie			
	Taille	Poids	TA	HU	Albumine	Sucre	*HIV*	Autre, à spécifier
CPN1								
CPN2								
CPN3								
CPN4								
Commentaires:								

Q44. Est-ce que les femmes sont informées des <u>types</u> de prélèvements lors de la remise du bulletin d'analyses (sang, urine, PV) ?

☐Oui ☐Non

Q45. Est-ce que les <u>conditions</u> de prélèvements leur sont clairement expliquées ?

☐Oui ☐Non

Si non, passez à la question suivante Q46

Si oui, cochez les conditions pour lesquelles elles reçoivent les informations appropriées :

Sang

- ☐ Nature du prélèvement
- ☐ A jeun, sauf en cas d'urgence
- ☐ Vérifier le traitement en cours
- ☐ Autre, à spécifier ……………………..

Urine

- ☐ Nature du prélèvement
- ☐ Urine ayant séjourné plus de 2 heures dans la vessie (ECBU)
- ☐ Vérifier le traitement en cours
- ☐ Autre, à spécifier ………………………………

Prélèvement Vaginal

- ☐ Nature du prélèvement
- ☐ Pas de rapport sexuel la veille
- ☐ Pas de traitement antibiotique en cours
- ☐ Pas de règles
- ☐ Pas de toilette intime, ni de traitement local
- ☐ Vérifier le traitement en cours
- ☐ Autre, à spécifier ……………………………………………

IV.BILAN DE LA CPN DE L'ANNE 2012

Q46. A quelle période de la grossesse, les femmes ont eu leur première CPN ?

Terme de la grossesse	Nombre de femmes	Total

1 –3 mois				
4 –6 mois				
7 –8 mois				
9 mois et plus				
Non déterminé				
Total				

Q47. Combien de femmes ont eu leur *premier contact* (la **<u>1ère</u>** *fois* que la femme se présente pour la consultation prénatale, qu'importe l'âge de la grossesse) ? _________ Femmes

Q48. Déterminez le nombre de femmes ayant bénéficié des visites 1 à 4 de Janvier à Avril 2012

	1 visite	2 visites	3 visites	4 visites	Total
Nombre					

Etude des facteurs socio-culturels et historiques limitant le recours au laboratoire dans les soins prénataux au Sénégal, Mali et Burkina Faso

Cette grille va nous permettre de répondre aux questions suivantes :

Quelle est la productivité du laboratoire en termes de tests de CPN par rapport aux ressources disponibles :

- *Ressources humaines : qualité et quantité*
- *Organisation interne*
- *Logistique : infrastructures, équipements, etc.*

Nom de la structure : ……………………………………….

Type de structure : ☐ Centre de santé ☐ EPS1 ☐ Clinique privée

Zone rurale ☐ Zone urbaine ☐

Durée de l'enquête : du _____ / ______ au _____ / ______

Prénoms et nom de l'enquêteur : ……………………………………………………………

Signature :

OBS : ……..

Prénoms et nom des participants à l'enquête :

Prénoms	Nom	Fonction	Téléphone	Mail
**1.**				
2.				
3.				
4.				
5.				
6.				
7.				
8.				

NB. Tous les coûts/prix sont exprimés en FCFA

	INDICATEURS			RESULTATS
**I .RESSOURCES HUMAINES**	Q1. Déterminez le profil et le nombre de prestataires en service au laboratoire ?			
	Profil	*Nombre*		Total
		Homme	Femme	
	Pharmacien /Médecin non spécialiste			
	Pharmacien/ Médecin Spécialiste			
	Ingénieur biomédical			
	Technicien supérieur			
	Technicien			
	Agent de santé communautaire (ASC)			
	Infirmier			
	Secrétaire			
	Technicien de surface			
	Personnel de nettoiement			
	Autre, à spécifier ……..................................... ………………………………………………. ………………………………………………			
	Total			
	Q2.Quel est le type, lieu et nombre d'années de formation du personnel du laboratoire?			
		Diplôme/Attestation/ Certificat	*Lieu de formation*	*Nombre d'années d'études*
	Pharmacien /Médecin non spécialiste			
	Pharmacien/ Médecin Spécialiste			
	Ingénieur biomédical			
	Technicien supérieur			
	Technicien			
	Agent de santé			

	communautaire (ASC)			
	Infirmier			
	Secrétaire			
	Autre, à spécifier ……........................			
**II.ORGANISATION**				
	**II.1COMMUNICATION**			
	**II.1.1COMMUNICATION INTERNE**			
	Q3. Tenez-vous des réunions internes ?		Oui	Non
	Q4. Si oui, quelle est la fréquence des réunions internes, cochez la (les) réponse (s)<br>☐ Hebdomadaire<br>☐ Mensuelle<br>☐ Trimestrielle<br>☐ Au besoin<br>☐ Autre, à préciser ……………………………………			
	Q5. Qui participe aux réunions internes ?<br>☐ Le personnel technique du laboratoire<br>☐ Le personnel externe au laboratoire,<br>☐ Autre, à spécifier<br>…………………………………………………………………………………………<br>……………………………………………………………………………………			
	Q6.Est-ce que le laboratoire produit un compte rendu d'activités (trimestriel/semestriel) ? (vérifier)		Oui	Non
	Q7. Utilisez-vous un Système de communication interne ; cochez la (les) réponse (s)<br>☐ Affiche<br>☐ PV de réunion<br>☐ Système intranet<br>☐ Connexion internet<br>☐ Autre, à préciser …………………………………………..			
	**II.1.2 COMMUNICATION EXTERNE**			
	**II.1.2.1 COMMUNICATION LABORATOIRE-PATIENTS**			
	Q8. Les prix des analyses sont-ils connus et visibles pour tous les		Oui	Non

	patients (CPN et autres) ? (Vérifier)		
	Q9. Quel est le délai de rendu des résultats aux patients ?<br>☐<24h ☐24-48h ☐>48h		
	Q10. Est-ce que le délai de rendu des résultats est le même pour toutes les analyses ?	Oui	Non
	Q11. Quelles sont les analyses, pour lesquelles les résultats sont rendus immédiatement ? Listez-les<br>…………………………. ………………………… ………………………<br>………………………… ………………………… ……………………….		
	Q12. Quel est le processus de rendu des résultats ?<br>☐ Sous plis fermés<br>☐ Sans enveloppe<br>☐ Autre, à spécifier ………………………………………………		
	Q13.Est ce que le processus de rendu des résultats garantit une confidentialité optimale ?	Oui	Non
	Q14. Si non dites pourquoi ? (Accessibilité, codification, nomination…)<br>--<br>--<br>--		
	Q15. Qui rend les résultats ?<br>☐ Le technicien de laboratoire<br>☐ L'infirmier<br>☐ La secrétaire<br>☐ L'assistant social<br>☐ Autre, à spécifier ………………………………..		
	Q16.Est-ce que le processus de rendu des résultats est le même pour toutes les analyses ?	Oui	Non
	Q17.Si non, quelles sont les analyses qui nécessitent une attention particulière ?<br>……………… ………………… ……………		

	Q18. Qui rend les résultats de la sérologie rétrovirale?<br>☐ Le technicien de laboratoire<br>☐ L'infirmier<br>☐ La secrétaire<br>☐ L'assistant social<br>☐ La sage femme ayant demandé l'analyse<br>☐ N'importe qu'elle sage femme<br>☐ Le responsable du laboratoire<br>☐ Autre, à spécifier ……………………………..		
	Q19. Est que les résultats positifs de la SRV sont rendus de la même manière que les résultats négatifs ?	Oui	Non
	Q20. Si oui, décrivez sommairement le rendu des résultats de la SRV *(passez à la question Q23)*<br>………………………………………………………………………………………<br>………………………………………………………………………………………<br>………………………………………………………………………………………<br>…………………………………………………………………………….		
	Q21.Si non, qu'est ce qui est prévu quand le résultat de la SRV est négatif ?<br>☐Les résultats des autres tests en plus de la SRV sont rendus par le laboratoire<br>☐Les résultats des autres tests en plus de la SRV sont rendus par la sage femme<br>☐Seul le résultat de la SRV est rendu par l'assistant social, le laboratoire rend le reste des tests<br>☐Autre, à spécifier …………………………………………………………………..		
	Q22.Si non, qu'est –ce qui est prévu quand le résultat de la SRV est positif ?<br>☐Seuls les résultats de la SRV sont rendus par *l'assistant social* avec *counceling* post test<br>☐*La sage femme* rend les résultats de la SRV avec *counceling* post test<br>☐ Les résultats des autres tests en plus de la SRV sont rendus par l'assistant social avec *counceling* post test		

	☐ Les résultats des autres tests en plus de la SRV sont rendus par la sage femme avec *counceling* post test<br>☐Le laboratoire rend directement tous les résultats<br>☐Autre, à spécifier …………………………………………………………………………		
	Q23. Quelle est l'heure de rendu des résultats ?<br>☐ Matin<br>☐ Après-midi<br>☐ A n'importe qu'elle heure<br>☐ Autre, à préciser ………………………		
	Q24. Quel est le délai de réception des prélèvements en provenance des autres unités<br>☐ Pas de délai<br>☐ Jusqu'à 10h<br>☐ Entre 10-12h<br>☐ Autre, à spécifier ……………………		
	Q25.Quel est le délai de réception des prélèvements en provenance de la maternité ?<br>☐ Pas de délai<br>☐ Jusqu'à 10h<br>☐ Entre 10-12h<br>☐ Autre, à spécifier ………………………..		
	Q26. Quels sont les horaires de fonctionnement de la salle de prélèvements ?<br>de ……à ………heures ?		
	**II.1.2.1 COMMUNICATION LABORATOIRE-STRUCTURE**		
	Q27. Est-ce que les recettes du laboratoire sont communiquées aux autres services lors des bilans annuels d'activités ?	Oui	Non
	Q28. Existe-t-il une confrontation entre le compte rendu du laboratoire et le bilan financier ?		
	Q29.Le Responsable du laboratoire est-il intégré dans l'équipe cadre de district/région ?		
	Q30.Est-ce que le laboratoire rend des résultats d'urgence aux prescripteurs, les sages femmes compris ?		

Q31.Si oui, quels sont les moyens de communication utilisés?

- ☐ Appel téléphonique
- ☐ SMS
- ☐ Mail
- ☐ Déplacement personnel
- ☐ Autre, à spécifier ……………………………………

Q32.Pouvez-vous estimer en 2012, le nombre de résultats communiqués, en urgence? …….

CPN	*Maternité*	*Les autres consultations*	*Total*

Q33. Estimez la durée des phases pré, ana et post analytiques des *tests rapides* réalisés au laboratoire

Tests	Durée phase pré-analytique (prélèvement et partie pratique) (min)	Durée phase analytique (réalisation du test -min.)	Durée phase post analytique (validation, enregistrement rendu résultats aux patients)
Albumine/Sucre			
TDR Paludisme			
HIV			

Q34. Les tests rapides sont-ils rendus au même moment que le reste du bilan prénatal ?

☐Oui ☐ Non

Q35. Estimez la durée des phases pré, ana et post analytiques des *tests prénataux suivants* réalisés au laboratoire ?

Tests	Durée phase pré-analytique (prélèvement acheminement (min)	Durée phase analytique (réalisation du test -min.)	Durée phase post analytique (validation, enregistrement rendu résultats aux patients)	Observations
GS/RH				
NFS				
TE				
BW				

II.2 ORGANISATION INTERNE

**II.2.1 FONCTIONNALITE DU LABORATOIRE**		
Q36. Est-ce que le laboratoire fonctionne 24h/24 ?	Oui	Non

Q37.Si oui, quel est le type de rotation du personnel ? ***(Si non passez à la question Q38)***

☐ Toutes les 6 heures ☐ Toutes les 8 heures ☐ Autre, à spécifier

Q38. Décrivez le système de rotation journalière (composition des équipes en termes de *qualification* et heures précises de rotation)

Equipes		*Heure de rotation*
1.		
2.		
3.		

**II.2.2. PRISE EN CHARGE DES PATIENTS**<br><br>Q39. Est-ce que les patients sont informés des conditions de prélèvements, au laboratoire ?	Oui	Non
Q40. Vérifiez- vous systématiquement si les conditions de prélèvements sont respectées avant tout prélèvement?		

Q41.Si oui, cochez les conditions préconisées, en fonction de la nature du prélèvement :

- Prélèvement de Sang :

☐ A jeun, sauf en cas d'urgence
☐ Vérifier le traitement en cours
☐ NA
☐ Autre, à spécifier ……………………………………

- Prélèvement d'Urine :

☐ Urine ayant séjourné plus de 2 heures dans la vessie (ECBU)
☐ Vérifier le traitement en cours
☐ NA
☐ Autre, à spécifier ………………………………………….

- Prélèvement Vaginal :

☐ Pas de rapport sexuel la veille
☐ Pas de traitement antibiotique en cours
☐ Pas de règles
☐ Pas de toilette intime, ni de traitement local
☐ Vérifier le traitement en cours
☐ NA
☐ Autre, à spécifier ………………………………………

Q42. Combien de femmes enceintes ont été reçues au laboratoire, pour un bilan prénatal complet :

Année	*2012*	*2013*	*2014(1er semestre)*
Interne			
Externe			
Total (%)			

Année	*2012*	*2013*	*2014(1er semestre)*
Interne			
Externe			
Total (%)			

Q43.Combien de PTME ont été réalisés au laboratoire ?

Q44.Quel a été la provenance des femmes reçues au laboratoire ?

	**PTME**				**Bilan prénatal**			
	CS	Privé	Hôp.	Poste	CS	Privé	Hôp.	Poste
2012								
2013								
2014(1er semestre)								
Total								

Q45. Faites le point entre la proposition, l'acceptation et le retrait des résultats de PTME, en 2012

	J	F	M	A	M	J	J	A	S	O	N	D	**Total**
P													
A													
E													
R													
**Ré(+)**													

	**P- Proposition; A-Acceptation; E- Effectué; R-Retrait ; Ré (+)- Résultats positifs**<br><br>Q46.Précisez la source de l'information (Q41)<br>☐ Registre PTME Laboratoire (☐ P; ☐A; ☐ E; ☐ R)<br>☐ Registre CPN (☐ P; ☐ A; ☐ E; ☐ R)<br>☐ Registre Laboratoire (☐ P; ☐ A; ☐ E; ☐ R)<br>☐ Registre service social (☐ P; ☐ A; ☐ E; ☐ R)<br>☐ Autre registre, à spécifier (☐ P; ☐ A; ☐ E; ☐ R) …………………………………………………
	Q47. Est-ce le laboratoire fait le bilan pré-opératoire ?<br>☐ Oui ☐ Non ☐ Non Applicable
	Q48.Si oui, spécifier les analyses demandées<br><br>…………………… ………………………… …………………………..<br>…………………… ………………………….. …………………………..
	**II.2.3. LOGISTIQUE**
	Q49. Où se fait le prélèvement des patients ?<br>☐ Dans les salles de manipulations<br>☐ Au niveau des couloirs<br>☐ Dans une salle dédiée<br>☐ Autre, à spécifier ………………………………………………………
	Q50.Quel est le mobilier existant dans la salle de prélèvements ?<br>☐ Au moins une chaise et une table pour le préleveur<br>☐ Au moins un fauteuil de prélèvements<br>☐ Au moins une table gynécologique pour les prélèvements vaginaux<br>☐ Au moins une lampe *Wood* pour les prélèvements vaginaux<br>☐ Autre, à spécifier ………………………………………………………

**III. ACTIVITES**	**III.1 ANALYSES**			
	Q51. Est-ce que les analyses suivantes sont réalisées au laboratoire ?	Oui	Valeur Référence, si applicable	Non
	− **Groupage Sanguin/Rhésus**		---------------	
	− **Glycémie à jeun**			
	− **Albumine /Sucre**		-----------------	
	− **Sérologie VIH**		-----------------	

	− **Test d'Emmel**		----------------	
	− **Sérologie syphilitique**		----------------	
	− NFS / Taux d'Hg			
	− Acide urique			
	− Sérologie toxoplasmose		--------------	
	− Sérologie rubéole		--------------	
	− Prélèvement Vaginal (PV) : *Streptococcus agalactiae*		--------------	
	Escherichia coli		--------------	
	Neisseria gonorrhoeae		--------------	
	Vaginose bactérienne		--------------	
	Candida albicans		--------------	
	− *Chlamydia trachomatis* (recherche directe)		--------------	
	− Mycoplasmes		-------------	
	− AgHBs		-------------	
	− Recherche d'agglutinines irrégulières		-------------	
	− Recherches de BAAR		--------------	
	**III.2 GESTION DE STOCK**			
	Q52. Y a t-il eu rupture de stock en 2012 ?	Oui		Non
	Q53. Si oui, pour quel (s) réactif (s) y a-t-il eu rupture ?	Quelle durée ? (jour, semaine, mois)		
	1.			
	2.			
	3.			
	4.			
	5.			
	6.			
	7.			
	8.			

**IV. EQUIPEMENTS**				

Q54.Quelle a été la fonctionnalité des équipements suivants en 2012 ?	Bonne	Moyenne	Défectueuse	Non applicable
– Automate d'Hématologie				
– Automate de Biochimie				
– Etuve bactériologique				
– Autoclave				
– Centrifugeuse				
– Microscopes optiques				
– Balances				
– Distillateurs				
– Autres, à spécifier….				

V. CONSOMMATION ENERGETIQUE ET HYDRAULIQUE

Q55. Quelle est la puissance annuelle de la consommation énergétique de la structure ?

	Puissance Maximale (PM)	Annuelle (=12x PM)
Total		

Q56.Quel est le montant annuel de la facture d'électricité de la structure ? ……………………………..

Q57. Faites la cartographie de tous les appareils installés au laboratoire et relevez leur puissance (Watt)

**Equipements**	**Marque**	**Puissance (Watt)**	**Durée de fonctionnement journalière**
**1.**			
**2.**			
**3.**			
**4.**			
**5.**			

6.			
7.			
8.			
9.			
10.			
11.			
12.			
13.			
14.			
15.			
16.			
17.			
18.			
19.			
20.			
21.			
22.			
23.			
24.			
25.			
26.			
27.			
28.			
29.			
30.			

Total		
Q58.Estimer la consommation moyenne annuelle en énergie au laboratoire (Comparer avec la grille finances) … ……… ……… ……… ……… ……… ……… ……… ………		

Q59. Combien de points d'eau existent au laboratoire ………………………………

Q60.Quelle est la consommation annuelle en eau ? (*Nombre de points d'eau x 0.1*m^3)= ………………………………..$m\,3$

Q61. Quel est le montant de la facture du laboratoire en eau ? *(à partir du coût du* m^3 *d'eau à la SDE (Comparer avec la grille finances si l'information est disponible)* ……………………………………………………………

……..

…….

CONCLUSION

Q62. Quelles appréciations faites-vous des services de CPN et de la maternité (remplissage du bulletin d'analyses, type d'analyses demandé, l'identification du prescripteur….) ?

Points forts	
Points faibles	
Recommandations	

Q63.Que pensez vous de votre statut au sein de la structure ?

Points forts	
Points faibles	
Recommandations	

Etude des facteurs socio-culturels et historiques limitant le recours au laboratoire dans les soins prénataux au Sénégal, Mali et Burkina Faso

Cette grille va nous permettre de répondre à la question suivante :

'Quelles sont les ressources mises en place au laboratoire par rapport aux autres services' ?

Nom de la structure : ……………………………………………………………………….

EPSI☐ Centre de Santé☐

Zone rurale☐ Zone urbaine ☐

Période de l'enquête : du _____ /______ au _____ /______ /2013

Prénoms et nom de l'enquêteur : ………………………………………………………….

Signature :

Prénoms et nom des participants à l'enquête :

Prénoms	Nom	Fonction	Téléphone	Mail
1.				
2.				
3.				
4.				
5.				

NB. Tous les coûts/prix sont exprimés en FCFA

I.RESSOURCES HUMAINES	
**INDICATEURS**	**RESULTATS**
Q1. Quel est le nombre de contractuels du laboratoire	
Q2. Quel est le nombre d'étatiques du laboratoire	
Q3. Quel est le nombre total de contractuels de la structure	

Q4. Quel est le nombre total d'étatiques de la structure	
**II. RECETTES**	
Q5. Quel est le budget de la structure	
Q6. Quel a été le budget d'exécution du laboratoire en 2012	
Q7. Quelles ont été les recettes de la structure sanitaire, en 2012	
Q8. Quelle a été la part du laboratoire dans les recettes de la structure sanitaire, en 2012	
Q9. Gestion financière des recettes : quelle part des recettes a été reversée au laboratoire, en 2012	

Q10. Est-ce que les recettes du laboratoire sont communiquées aux autres services

□oui □non

Si oui à quelles occasions

- □ Lors des bilans annuels
- □ Dans les rapports d'activités
- □ Lors des réunions de coordination
- □ Lors des réunions de staff
- □ Autre, à spécifier ____

Q11. Qui décide des prix des analyses prénatales ?

- □ Le niveau central
- □ Le comité de santé
- □ Le Médecin Chef de District (MCD)
- □ Le Conseil d'Administration (CA)
- □ L'Equipe Cadre de District (ECD)
- □ Autre, à spécifier …………………………………………………

Q12. Sur quelles bases les prix sont établis ?

- □ Sur la base des B
- □ Sur la base des minima et maxima
- □ Sur la base des recettes
- □ Sur autre base, à spécifier ………………………………………………..

Q13. Gestion des cas sociaux : donner le nombre et type de gratuité enregistrés en 2012 au laboratoire

	Femmes enceintes pour le *Bilan prénatal*	Autres patients	Total
Quel est le nombre ?			

Quel type de gratuité?	100%			
	50%			
	25%			

Q14. Quelles ont été les recettes dans les services en 2012 ?

Service	Montant
Laboratoire	
Maternité	
Médecine	
Ophtalmologie	
ORL	
Orthopédie	
Pédiatrie	
Radiologie	
SAU	
Stomatologie	
Pharmacie	
Autre, à spécifier …………………………………… ……………………………………………… ……………………………………………… ……………………………………………	
TOTAL	

III.DEPENSES

III.1 DEPENSES DES SERVICES

Q15. Quelles ont été les dépenses dans les services en 2012 ?

**Service**	**Montant**

Laboratoire	
Maternité	
Médecine	
Ophtalmologie	
ORL	
Orthopédie	
Pédiatrie	
Radiologie	
SAU	
Stomatologie	
Pharmacie	
Autre, à<br>spécifier ………………………………………<br>……………………………………………………<br>……………………………………………………<br>……………………………………………………	
TOTAL	

Q16. Quelles ont été les dépenses en 2012 pour les tests suivants ?

	**Nombre de kits**	**Coût unitaire**	**Cout total (FCFA)**
**BW**			
- RPR			
- TPHA			
**Albumine/sucre**			
**NFS**			

− Lyse			
− Diluent			
− Rinse			
− Sang de contrôle			
− Autre, à spécifier … …………….			

III.2 PERSONNEL CONTRACTUEL

Q17.Quel est le salaire mensuel du personnel contractuel en fonction de la charge de travail ?

Hiérarchie	Nombre	Charge horaire	Salaire mensuel
Pharmacien / Médecin non spécialiste			
Pharmacien/ Médecin Spécialiste			
Technicien supérieur			
Technicien			
Secrétaire			
Personnel de nettoiement			
Maitresse sage femme			
Sages femmes			
Autre, à spécifier ……............ .... ……………....			

Q18.Quelles sont les autres charges salariales associées au personnel auxiliaire ? ____

Q19. Quelles sont les charges patronales supportées par la structure pour tout le personnel contractuel (IPRES, motivations, gardes etc) ? _____________________________

Q20. Quelle est la périodicité du paiement des motivations pour le personnel étatique ?

☐ mensuel ☐ trimestriel ☐ semestriel ☐ annuel ☐ non déterminé

Q21. A quand remonte le paiement des dernières motivations ? Le ______/ ______ /_______

Q22. Motivations mensuelles pour les prestataires suivants :

- ☐ Le chirurgien dentiste _______________
- ☐ Le gynécologue-obstétricien __________
- ☐ Biologiste _________________________
- ☐ Technicien supérieur ________________
- ☐ Technicien de laboratoire ____________
- ☐ Maitresse sage-femme _____________
- ☐ Sage femme ____________________

Q23.Comment se fait la motivation du personnel contractuel?

- ☐ 20% des recettes
- ☐ 25% des recettes
- ☐ Autre, à préciser ……………………..

III.2 PERSONNEL ETATIQUE

Q24. Quel est le salaire mensuel du personnel étatique en fonction de la charge de travail ?

Hiérarchie	Nb	Charge horaire	Salaire mensuel	Mairie (Mairie) ou Ministère (Min)
Pharmacien / Médecin non spécialiste				
Pharmacien/ Médecin Spécialiste				
Technicien supérieur				
Technicien				
Secrétaire				
Personnel de nettoiement				
Autre, à spécifier ……............				

…………………………				

Q25. Quelles sont les charges patronales supportées par la structure pour le personnel étatique (motivations, gardes etc.) ? ________________________

Q26. Quelles sont les charges patronales supportées par la structure pour tout le personnel contractuel (IPRES, motivations, gardes etc) ? ____________________________

Q27. Quelle est la périodicité du paiement des motivations pour le personnel étatique ?

- mensuel
- trimestriel
- semestriel
- annuel
- non déterminé

Q28. A quand remonte le paiement des dernières motivations ? Le _____/ _____/______

Q29. Motivations mensuelles pour les prestataires suivants :

- Le chirurgien dentiste _______________
- Le gynécologue-obstétricien __________
- Biologiste _________________________
- Technicien supérieur _______________
- Technicien de laboratoire ___________
- Maitresse sage-femme _____________
- Sage femme ______________________

Q30.Comment se fait la motivation du personnel contractuel?

- 20% des recettes
- 25% des recettes
- Autre, à préciser ……………………..

III.2 DEPENSES EN 2012

Q31.Quelles ont été les dépenses, en 2012, pour les rubriques suivantes ?

	Laboratoire	Autres services	Total
Réactifs et consommables			
Eau			

Electricité			
Gaz			
Téléphone			
Maintenance des équipements			
Autres, à préciser			

III.3AUTRES INVESTISSEMENTS : EN 2012

Q32.Quel a été le coût annuel des dépenses, en 2012 pour les activités suivantes ?

	Laboratoire	Autres services	Total
Achats d'équipements			
Réhabilitation/construction des locaux			
Formation du personnel			
Autres, à spécifier			

IV. COUT DES PRESTATIONS

Q33. Est-ce que les analyses suivantes sont réalisées au laboratoire ? (*prendre la liste des prestations et couts associés)*

	oui	non	coût
Groupage Sanguin/Rhésus			
Glycémie à jeun			
Albumine/sucre			

Test d'Emmel			
NFS			
Sérologie VIH			
Sérologie toxoplasmose			
Sérologie rubéole			
Sérologie syphilitique			
Acide urique			
Créatinine			
Prélèvement Vaginal (PV) : recherche de germes banals			
Chlamydia trachomatis (recherche directe)			
Mycoplasmes			
AgHBs			
Recherche d'agglutinines irrégulières			
Tests de Coombs			
TDR Paludisme			
Goutte épaisse			
TB			
Q34. Quel(s) est (sont) le (s) coût(s) global (aux)du bilan standard prescrit par la structure ?<br>………………………….FCFA ; ………………………………….FCFA			

V. SOURCES DE FINANCEMENT ET MONTANTS ASSOCIES ?

Q35. Quelles sont les sources de financement et montants associés ?

Types	Montant en 2012	%
☐ Recettes propres		
☐ Subventions états		
☐ Dons et legs		

□ Autre, à préciser ……………		
Total		

VI.1. CENTRES DE SANTE

Q36.Existe t-il un comité de santé ? □ Oui □ Non

Q37. Si oui, à quelle date a-t-elle- été créée ? : ________ /_________/ _________

Q38.Quelle est la composition du bureau ?

	Formation initiale	Expériences professionnelles	Durée dans le poste	Activités parallèles
Président				
Vice président				
Trésorier				
Adjoint trésorier				
Commissaires aux comptes				
1.				
2.				
Autre, à spécifier ……… ………………………				

Q39. Décrivez les activités du comité de santé

VI.2.ETABLISSEMENT PUBLIQUE DE SANTE (EPS 1)

Q40. Quelle est la date de création de l'EPS ? ___________ /___________/ _____________

Q41. Quelle est la durée du mandat des membres du Conseil d'Administration ?

- ☐ 3 ans
- ☐ 2 ans
- ☐ Autre, à préciser …………………………………………..

Q42. Quelle est la composition du bureau du CA ?

**Bureau**	**Nombre**
☐ Président du conseil régional	
☐ Mairie de la commune	
☐ Président de la Commission Médicale d'Etablissement	
☐ Représentant du Ministère de la Santé et de l'Action Sociale	
☐ Représentant des usagers	
☐ Représentant du personnel	
☐ Représentant Organisme de Prévoyance Sociale	
☐ Représentant du Ministère de l'Economie et des Finances	
☐ Personnes qualifiées	
Autres, à préciser ……………………. ………………………………………………… ……………………………………………………………………….	
Total	

Q43.Quelles sont les zones d'interventions du CA ?

- ☐ Recrutement personnel
- ☐ Coût des prestations
- ☐ Achat de matériel
- ☐ Infrastructures
- ☐ Paquet de services
- ☐ Autre, à spécifier ……………………………………………………………

Q44.Quelle est la périodicité des réunions du CA ?

☐Mensuel ☐Trimestriel ☐Semestriel ☐Au besoin

□Autre, à spécifier …………………………………………………………….

Q45. Quelles ont été les avantages du changement de statut c'est-à-dire du CS à l'EPS1

	Rubriques	*Résultats*
Structure	− Renforcement du plateau technique	□ oui □ non
	− Augmentation du budget	□ oui □ non
	− Création de nouveaux services	□ oui □ non
	− Augmentation des recettes	□ oui □ non
	− Augmentation des motivations financières	□ oui □ non
Laboratoire	− Recrutement de personnel qualifié	□ oui □ non
	− Augmentation des prestations	□ oui □ non
	− Augmentation des recettes	□ oui □ non
